# Statistical image properties predict aesthetic ratings in abstract paintings created by neural style transfer

**DOI:** 10.3389/fnins.2022.999720

**Published:** 2022-10-13

**Authors:** Hannah Alexa Geller, Ralf Bartho, Katja Thömmes, Christoph Redies

**Affiliations:** Experimental Aesthetics Group, Institute of Anatomy I, Jena University Hospital, Friedrich Schiller University Jena, Jena, Germany

**Keywords:** experimental aesthetics, artificial intelligence, visual artwork, neural style transfer (NST), abstract art, art style, traditional art, aesthetic terms

## Abstract

Artificial intelligence has emerged as a powerful computational tool to create artworks. One application is Neural Style Transfer, which allows to transfer the style of one image, such as a painting, onto the content of another image, such as a photograph. In the present study, we ask how Neural Style Transfer affects objective image properties and how beholders perceive the novel (style-transferred) stimuli. In order to focus on the subjective perception of artistic style, we minimized the confounding effect of cognitive processing by eliminating all representational content from the input images. To this aim, we transferred the styles of 25 diverse abstract paintings onto 150 colored random-phase patterns with six different Fourier spectral slopes. This procedure resulted in 150 style-transferred stimuli. We then computed eight statistical image properties (complexity, self-similarity, edge-orientation entropy, variances of neural network features, and color statistics) for each image. In a rating study, we asked participants to evaluate the images along three aesthetic dimensions (Pleasing, Harmonious, and Interesting). Results demonstrate that not only objective image properties, but also subjective aesthetic preferences transferred from the original artworks onto the style-transferred images. The image properties of the style-transferred images explain 50 – 69% of the variance in the ratings. In the multidimensional space of statistical image properties, participants considered style-transferred images to be more Pleasing and Interesting if they were closer to a “sweet spot” where traditional Western paintings (JenAesthetics dataset) are represented. We conclude that NST is a useful tool to create novel artistic stimuli that preserve the image properties of the input style images. In the novel stimuli, we found a strong relationship between statistical image properties and subjective ratings, suggesting a prominent role of perceptual processing in the aesthetic evaluation of abstract images.

## Introduction

The question of whether computers can create artworks has intrigued computer scientists and artists alike (Hertzmann, 2018; Lomas, 2018; Mazzone and Elgammal, 2019; So, 2020; Cetinic and She, 2022). In the art world, the usage of computers has been a research subject for more than 50 years (Giloth and Pocock-Williams, 1990; for a review, see Nake, 2012). After decades of relative quiescence, artificial intelligence (AI) has taken the art world by storm. A key trigger of this recent development was the introduction of Convolutional Neural Networks (CNNs), which have gained enormous popularity, in part because of their highly effective application in computer vision (LeCun et al., 2015). CNNs are neural networks with convolutional layers, which are particularly well suited for processing images. Under supervised training with more than a million stimuli, they can achieve extraordinarily high (human-like) accuracy rates, for example, in recognizing large series of natural objects and scenes (LeCun et al., 2015). Low- and intermediate-level responses of the network resemble those recorded in the early human visual system (Krizhevsky et al., 2012; Yosinsky et al., 2014; Güçlü and van Gerven, 2015; Cadena et al., 2019; Kindel et al., 2019). At higher levels, feature responses integrate over larger input regions to represent increasingly more complex (parts of) objects, similar to neural responses in extrastriate cortical regions (Cadieu et al., 2014; Yamins et al., 2014).

At present, an increasing number of artists are experimenting with computer-assisted art creation and automation in their work. The most widely used approach to generating art is based on a type of CNN called Generative Adversarial Networks (GANs; Goodfellow et al., 2014), as well as their advancements, such as AI Creative Adversarial Networks (AICANs; Elgammal et al., 2017). These developments give rise to questions about ethics, authenticity, and autonomy as well as to philosophical controversies regarding creativity and artistry (Mazzone and Elgammal, 2019; So, 2020; Cetinic and She, 2022).

Neural Style Transfer (NST; Gatys et al., 2015) represents another way of how CNNs have found their way into the art world. By applying NST, the color and texture information of one input image [termed *style image* by Gatys et al. (2015)] can be transferred onto another input image [termed *content image* by Gatys et al. (2015)], thus generating a novel style-transferred output image (So, 2020). Artists and scientists have widely used these algorithms to generate artworks and experimental stimuli (for reviews, see Semmo et al., 2017; Jing et al., 2020; So, 2020; Santos et al., 2021; Zhang et al., 2021). In recent years, many different NST algorithms have been published with distinct properties, features and performance. Note that the meaning of the term “style” in NST differs from its definition in art history or art theory. In NST, style refers to the perceptual texture of a single artwork, which is represented in a feature space designed to capture texture information (Gatys et al., 2016). In the present study, we use the term in this sense. By contrast, artistic style can be defined as the style of a particular artist or school or movement. For example, Davis (2011) uses the term “style” to denote specific pictorial configurations that stem from the artwork being of a particular origin. Style analysis (“stylometry”) allows art experts, for example, to identify the artist of an artwork. Style identification can be assisted by computers, utilizing CNNs for instance (Wallraven et al., 2009; Graham et al., 2012; Van Noord et al., 2015; Chu and Wu, 2018).

Neural style transfer (NST) facilitates the creation of large numbers of artworks for statistical analysis and experimental investigations. However, the use of NST-generated stimuli for aesthetic research has several shortcomings. (1) Although the computational paradigms underlying NST are relatively well defined and understood (Semmo et al., 2017; Kotovenko et al., 2019; Hien et al., 2021), it is less well known how objective (physical) image properties are modulated by NST and how they mediate the aesthetic attributes and the liking of the generated images (Zhang et al., 2021). (2) The responses of beholders may be biased against computer-generated art (Chamberlain et al., 2018). (3) There is a debate of whether artificial intelligence can create artworks at all (for a review, see Cetinic and She, 2022). For example, Hertzmann (2019) reasoned that computers cannot be credited with authorship of artworks, but they can assist artists and serve as an engine for innovation. Similarly, McCormack et al. (2019) contest that computers can have artistic creativity and autonomy. Taking an opposite viewpoint, Mazzone and Elgammal (2019) claimed that they succeeded in developing an almost autonomous computer algorithm that is capable of producing artworks.

The present study is an attempt to shed more light on computer-generated art. Using NST, we created a set of artificial abstract artworks and analyzed their perceptual structure by calculating statistical image properties (SIPs) that have been associated previously with aesthetic perception and affective images (Braun et al., 2013; Brachmann et al., 2017; Redies and Brachmann, 2017; Grebenkina et al., 2018; Redies et al., 2020; see also Supplementary material for a comprehensive description of the SIPs used in the present study). In a behavioral experiment, we investigated how the SIPs relate to subjective aesthetic ratings.

It is generally accepted that aesthetic ratings depend not only on perceptual processing, but also on cognitive processing and emotional attributes of images (Jacobsen, 2006; Chatterjee and Vartanian, 2014; Graf and Landwehr, 2015; Redies, 2015). Cognitive and emotional factors may potentially modify or confound aesthetic responses to perceptual features of visual stimuli, such as the SIPs. Therefore, in line with our focus on perceptual factors, we minimized the effects of cognitive and emotional processing in the present study by using abstract (non-figurative) stimuli. We combined 25 abstract artworks from different artists and diverse art styles (that served as *style images* for NST; Gatys et al., 2015; see Supplementary Table 1) with 150 random-phase images (*content images* for NST; Gatys et al., 2015) to generate 150 novel style-transferred images. Note that our *content images* for NST did not display any recognizable content. In the following, we will therefore refer to them as *random-phase images*.

Aesthetic ratings can be defined along different dimensions. Berlyne (1970) asked participants to describe artworks in terms of *pleasingness* and *interestingness*. The two terms correlated with other rating terms, such as *complexity* and *novelty*, to different degrees. Augustin et al. (2012) found that for different image categories, including artworks, landscapes, and faces, participants use different sets of aesthetic terms to describe them. Lyssenko et al. (2016) studied the qualitative descriptions of abstract artworks and identified both descriptive, image-related terms (for example, *structured, colorful*, and *dark*) and affective terms (for example, *happy*, *boring*, and *warm*). Marković and Radonjić (2008) established four subjective dimensions of the aesthetic experience of paintings, which represent the main psychological and behavioral domains: Hedonic Tone and Relaxation (affective or emotional), Regularity (perceptual or cognitive), and Arousal (motivational). For our study, we chose rating dimensions for each of these domains to cover a wide range of the aesthetic experience: Pleasing (Hedonic Tone), Harmonious (Regularity), and Interesting (Arousal). The aesthetic scales used in the present study were previously shown to correlate with image properties (Schwabe et al., 2018; Stanischewski et al., 2020), and they have been associated with different aspects of aesthetic perception and evaluation (Cupchik and Gebotys, 1990; Marković, 2012; Graf and Landwehr, 2015).

As shown before, the SIPs of abstract or modern artworks overlap to a large extent with those of traditional artworks of different cultural provenance, but particular subtypes of modern art can also deviate substantially from traditional art (Redies and Brachmann, 2017; Mather, 2018). We therefore compare the artificially created artworks with a set of 1629 traditional Western paintings (JenAesthetics dataset; Amirshahi et al., 2015). This dataset comprises diverse artworks from different periods, styles, artists, and depicted subject matters. We also investigate how and if this comparison can be related to the aesthetic ratings of our style-transferred images.

Individuals share common aesthetic taste, but they also show individual preferences. The proportion of private taste versus shared taste varies according to the type of images viewed (Leder et al., 2016; Vessel et al., 2018). Some of the differences in private taste for artworks can be related to differences in the personality traits of the beholders, for example, openness to experience (Chamorro-Premuzic, 2009). Interestingly, the subjective interpretation of the rating terms by individual beholders also depends on personality traits (Lyssenko et al., 2016). In view of these previous results, we also clustered participants and analyzed their results separately.

The purpose of the present study is to address the following research questions: (1) In an exploratory analysis, we compare the SIPs of the input images (original artworks and random-phase images) with their style-transferred derivatives to find out how well NST transfers SIPs. (2) We investigate whether NST transfers participants’ subjective ratings from the two types of input images to the style-transferred (output) images. This analysis was also done for clusters of participants. We hypothesize that the rating responses are largely driven by the style of the original paintings, and that, as a consequence, preference for a particular style is transferred from the original abstract artworks onto their style-transferred counterparts. (3) Furthermore, we were interested in how well the SIPs can predict the aesthetic ratings of the style-transferred images. (4) We compare the artificially created artworks with the JenAesthetics dataset. We hypothesize that style-transferred images prompt higher aesthetic responses in the beholders if the values for the SIPs of the style-transferred images are closer to those of traditional artworks.

## Materials and methods

### Stimuli

**FIGURE 1:**
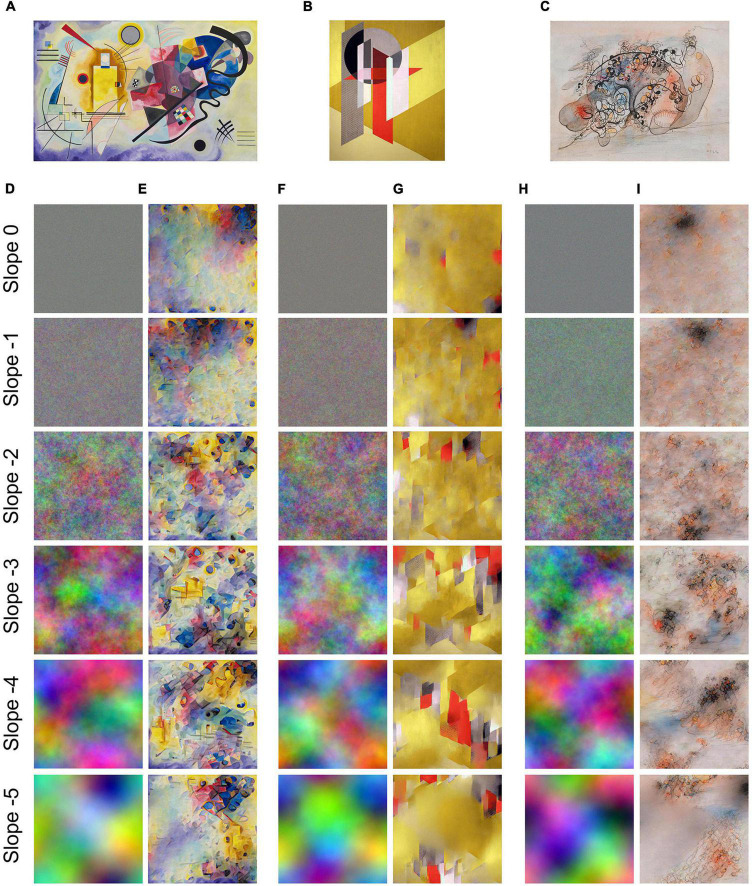
Examples of the three image categories studied. Original artworks **(A–C)** are shown on top of the random-phase images **(D,F,H)** that were used to generate the style-transferred images **(E,G,I)**, respectively. The slope of the log-log plots of Fourier power *vs.* spatial frequency is indicated on the left-hand side of each row. Original artworks are **(A)** *Gelb-Rot-Blau* by Wassily Kandinsky (1925); **(B)** *Z VII* by László Moholy-Nagy (1926); and **(C)** *Untitled* by WOLS, ca. 1940.

We used three different types of stimuli. First, we selected 25 abstract artworks by different artists. Care was taken to include paintings from diverse abstract art styles, including Abstract Expressionism, Art Informel, Color Field Painting, Constructivism, Dadaism, Hard-Edge Painting, Monochrome Painting, Neo-Expressionism, Op Art, Orphism, and Tachism. Most of the images were from a dataset used in previous studies (Mallon et al., 2014; Lyssenko et al., 2016). Two additional images were downloaded from the internet. The artists and information on the paintings are listed in Supplementary Table 1. Example paintings are shown in Figures 1A–C.

Second, we generated a set of 150 random-phase images with different Fourier spectral properties (for examples, see Figures 1D,F,H; Simoncelli and Olshausen, 2001; Galerne et al., 2010). Grayscale random-phase images can be generated easily and in great numbers for different slopes in log-log plots of Fourier power versus spatial frequency (Spehar et al., 2016). The random-phase patterns with different spectral slopes vary in their relation of fine detail and coarse image structure. Because the neural network used by the NST algorithm is trained on colored images and color is an important attribute of aesthetic judgments, we decided to generate colored versions of the random-phase images (Galerne et al., 2010). Colored versions of the random-phase patterns were obtained by merging different grayscale images of the same slope in the three channels of the RGB color space. In the present study, random-phase patterns had Fourier slopes that ranged from –5 to 0 in increments of 1 (–5, –4, –3, –2, –1, and 0). For each slope, 25 images were created. The images had a resolution of 1024 × 1024 pixels.

Third, we generated 150 images with NST (for examples, see Figures 1E,G,I). Each of the 25 styles of the original paintings was transferred onto 6 colored random-phase images with the different slopes (see above). Each style transfer was based on a different random-phase image. We used a revised version of the Style Transfer by Relaxed Optimal Transport and Self-Similarity (STROTSS) algorithm by Kolkin et al. (2019)^1^. The reasons for choosing this neural style transfer method were the availability of verified code, the speed of the method and the ability to produce images at a relatively high resolution (1024 × 1024 pixels). In addition, STROTSS is an optimization-based style transfer method that produces similar quality images for different styles and content. The parameter settings were identical to those used by Kolkin et al. (2019).

For the rating experiment, the stimuli were displayed on a ColorEdge CG241W screen (Eizo, Hakusan, Japan) in a darkened environment. A viewing distance of 80 cm was secured using a chin rest, resulting in a viewing angle of 20° for the target stimuli that were presented at 28.22 cm × 28.22 cm (800 × 800 pixels). The monitor was calibrated with an i1 Display pro calibrator (X-Rite, Grand Rapids, MI, U.S.A.; settings, brightness 120cd/m^2^; white point D65; gamma, 1.0 for all RGB channels).

### Participants

Forty volunteers (14 male and 26 female) participated in the rating experiment at Jena University Hospital. The duration of the experiment was about 60 min. Participants were paid €8 for taking part in the rating study. The mean age of the participants was 23 (range 18 to 30) years. One participant reported left-handedness, the remaining 39 were right-handed. In a short questionnaire on art interest, applied at the beginning of the experiment, one participant reported no interest in art, 13 participants reported being somewhat interested, and 26 participants reported an interest in art. Sixteen study participants had a medical background (mostly medical students), eleven studied history of art and film studies, the remaining 13 were university students from various other fields, such as economy, law, or chemistry.

The study was designed according to the specifications of the World Medical Association Declaration of Helsinki and approved by the Ethics Committee of Jena University Hospital (approval no. 2021-2223-Bef). The participants gave their written informed consent prior to the experiment. They were informed that they can freely withdraw from the experiment at any time without any repercussions.

### Procedure

Prior to the experiment, the participants were presented with a sheet of instructions for the experiment. Moreover, the participants were asked to answer a few demographic questions (age, gender, profession/field of study, level of interest in art, vision impairment and handedness). After completing the short questionnaire, the experiment was launched in full screen (1920 × 1200 pixels). Participants were asked to complete a short test-like run to familiarize them with the experimental procedure and the rating scale. For this supervised run, unrelated figurative paintings were used.

**FIGURE 2:**
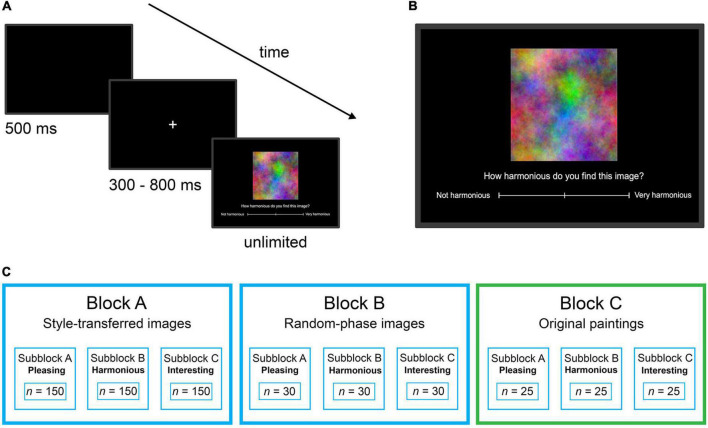
Experimental procedure. The schedule is shown in **(A)** with the presentation times indicated below each screen shot. **(B)** Shows a magnification of the screen display where ratings are entered by a mouse click on the scale below the image. **(C)** Illustrates the sequence of the rating blocks. Within the blue boxes, images and block sequences were randomized while the green box indicates a fixed position. *n*, number of images.

The experiment was divided into three blocks, one for each of the three image categories (abstract paintings, random-phase images, and style-transferred images; Figure 2C). Each of these main blocks consisted of three sub-blocks for the three rating dimensions (Pleasing, Harmonious, and Interesting). The experiment started in a randomized order with either the random-phase image block or the style-transferred image block (Figure 2C). The abstract paintings were always presented as the final block so that the participants’ ratings of the style-transferred images were not influenced by the original paintings. A disadvantage of this schedule is that the first two blocks possibly affect the ratings of the last block (original paintings). All 40 participants rated all 25 abstract paintings and all 150 style transfers. To avoid screen fatigue, every participant rated only 30 out of the 150 random-phase images (balanced with respect to their Fourier slope), resulting in 8 ratings per random-phase image. Within all main blocks, the order of the sub-blocks was randomized as was the image sequence within all sub-blocks (Figure 2C). In between blocks and sub-blocks, participants were allowed to take an optional break.

Within each trial, first, a blank black screen was presented for 500 ms followed by a white fixation cross, which appeared for a random duration between 300 and 800 ms (Figure 2A). Then, the target image was presented on the same black background alongside with a continuous rating scale below the image (Figure 2B). The rating scale for Harmonious ranged from “not harmonious” to “very harmonious.” The other ratings scales (Pleasing, Interesting) were presented in an analogous manner. Viewing time was not limited, but when participants entered the response by clicking on the scale using the computer mouse, the next trial was initiated. Median response time was 2.1 s (interquartile range: 1.6–3.0 s) with no difference between the image categories. The code for the presentation procedure was based on PsychoPy (Peirce, 2009).

### Statistical image properties

Aesthetic ratings by human observers correlate with statistical image properties (SIPs; see Introduction section). Previous studies indicated that SIPs can overlap to a large degree in their predictive power for aesthetic ratings (for example, see Redies et al., 2020), possibly because many of these SIPs cover similar aspects of image structure (Braun et al., 2013; Van Geert and Wagemans, 2020). Consequently, the SIPs do not predict aesthetic ratings independently of each other, which can cause problems with multicollinearity in multiple linear regression analysis. Therefore, we needed a set of SIPs that showed as little overlap as possible while still covering the multidimensional SIP space well.

Our starting point was a set of 29 SIPs (calculated at a resolution of 800 × 800 pixels), which are described in detail in the Supplementary material. An exploratory principal component analysis (PCA) with the 29 SIPs revealed that each of the three image categories can be described by a different combination of the variables, confirming the usefulness of the variables in describing images with different structural characteristics. For the subsequent analyses, we reduced the initial set of 29 SIPs to eight largely independent SIPs (Table 1) by pursuing the following strategies:

**TABLE 1:** Statistical image properties used in the analysis.

Abbreviation	Description	Correlation with (Spearman’s coefficient ρ)^1^	References
Complexity	Mean of all gradient strengths in the gradient image (HOG approach)	Fourier slope, 0.70; P_f_(2), –0.63; P_f_(4), –0.67; P_a_(8), –0.60; P_a_(16), –0.64; P_a_(30), –0.67; HSV (V) entropy, 0.63	Braun et al. (2013)
Self-similarity	Similarity of the histograms of oriented gradients for a subregion of an image, compared to the histogram of the entire image (PHOGs approach)	symmetry lrud^2^, 0.72; P_f_(8), –0.68; P_f_(16), –0.63	Amirshahi et al. (2012)
2^nd^-order entropy	2^nd^-order entropy based on pairwise statistics of edge orientations across an image	PHOG anisotropy, –0.72; 1st-order entropy, 0.84	Redies et al. (2017)
Variance P_a_(2)	Total variance over all CNN filter entries of 2 × 2 subregions at convolutional layer 1 (inverse measure of richness)	P_a_(4), 0.92; P_a_(8), 0.83; P_a_(16), 0.77; P_a_(30), 0.70; HSV (S) entropy, –0.71; HSV (V) entropy, –0.70	Brachmann et al. (2017)
Variance P_f_(30)	Median over the variances of each of the CNN filter entries for 30 × 30 subregions at convolutional layer 1 (measure of variability)	P_f_(8), 0.63; P_f_(16), 0.86; symmetry lrud^2^, –0.61; HSV (S) entropy, 0.60	Brachmann et al. (2017)
Lab (b)	Mean value for the b (yellow-blue) channel of the L*a*b* color space	Lab (L), 0.62; HSV (H), –0.64; HSV (V), 0.62	Li and Chen (2009); Mallon et al. (2014)
HSV (S)	Mean value for the S (saturation) channel of the HSV color space	P_a_(4), 0.63; P_a_(8), 0.65; P_a_(16), 0.69; P_a_(30), 0.73	Li and Chen (2009); Mallon et al. (2014)
HSV (H) entropy	Shannon entropy of the histogram of values for the H (hue) channel of the HSV color space (colorfulness)	HSV (S) entropy, 0.83	

^1^Correlations are listed if the Spearman coefficient |ρ| is larger than 0.60 for the style-transferred images.

^2^Combined mirror symmetry (left/right and up/down).

**FIGURE 3:**
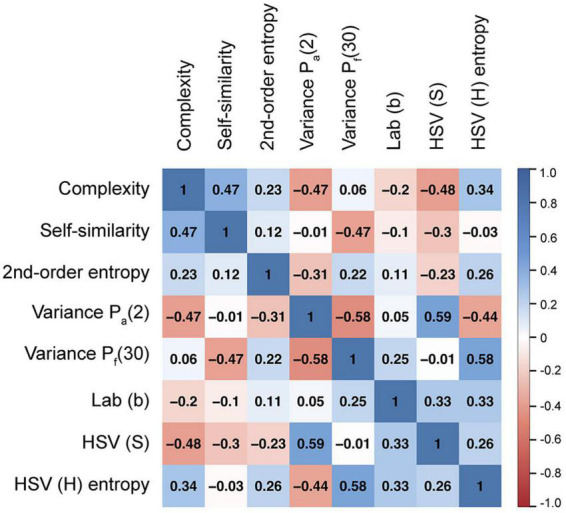
Correlation matrix for the eight SIPs that were investigated. The numbers represent the Spearman’s coefficients ρ that were calculated for the 150 style-transferred images. The color indicates positive (*blue*) and negative (*red*) correlations. The shading represents the strength of the correlations, with darker shadings representing stronger correlations (see color bar).

(1) We decreased multicollinearity between the 29 variables (SIPs) by regression subset selection. To this aim, we performed an exhaustive search for the subset of SIPs that best predicts the three rating dimensions for the 150 style-transferred images. Regression subset selection was accomplished with the leaps package of the R project (Miller, 1990). The leaps package returned the 10 best models (i.e., models with the highest *R^2^_adj_* values) for all possible model sizes (one to 29 predictive variables). The output graphs indicate how often a given variable is predictor in the different models. Based on these results, we selected the twelve variables that predicted the ratings most robustly across different models, for at least one of the rating dimensions.
(2) We then calculated a correlation matrix for the twelve remaining SIPs. Spearman’s rank (non-parametric) correlation coefficients ρ were used as many SIPs were not normally distributed. We eliminated another four SIPs which showed relatively high correlations with other SIPs (ρ > 0.6). Figure 3 lists the Spearman coefficients of the correlations between the eight remaining variables. They reflect the complexity and distribution of luminance and color gradients, and features derived from the CIELab and HSV color spaces (Table 1).

(3) The predictive power of the eight remaining variables and their large degree of independence was confirmed by calculating coefficients of determination (*R*^2^) in multiple linear regression models. *R*^2^ values were adjusted to account for the number of predictors and the number of datapoints (*R^2^_adj_*). The *R^2^_adj_* values in the final (reduced) model with eight variables (Supplementary Table 2) were of similar magnitude as the *R^2^_adj_* values in a model comprising the first eight principal components (PCs) of the 29 original variables (see Supplementary Table 2). This result suggests that much of the predictive power was preserved in the final model.

We exploratively plotted another regression subset selection for the remaining eight variables for all image categories and all rating dimensions (leaps function of R statistics; R Development Core Team, 2017). It reveals that our variable selection consistently predicts the ratings for one or more of the three image categories (see Supplementary Figure 1).

### Statistical methods

For statistical analyses, we used the R program (R Development Core Team, 2017) and PRISM for macOS, version 8.4.3 (GraphPad Software, San Diego, CA, U.S.A.). To compare multiple median values, we used the (non-parametric) Kruskal-Wallis test because most SIPs were not normally distributed. Subsequently, Dunn’s post-test was applied to obtain multiplicity-adjusted *p*-values for pairwise comparisons.

For the β* values, we use the following definitions for the size of the observed effects (Acock, 2014): | β*| < 0.2, weak effect; 0.2 ≤ | β*| < 0.5, moderate effect; and | β*| ≥ 0.5, strong effect. The same scheme was used to describe the strength of Spearman correlations. In the Figures and Tables, β* values for variables with asterisks had a significant effect on the ratings when the other variables were controlled for in the respective models.

As a measure for the distance between a given image and the JenAesthetics dataset of paintings in the multidimensional space of SIPs, we calculated the squared Mahalanobis distance with the *mahalanobis* program in the *stats* package of R statistics (R Development Core Team, 2017). This measure is a multivariate equivalent of the Euclidean distance and takes the full covariance matrix into account.

The participants were clustered according to how they evaluated images along the three rating dimensions Pleasing, Harmonious, and Interesting. K-means clustering was carried out with the *kmeans* program of R statistics (R Development Core Team, 2017). The clustering of participants was based on: (1) the correlations between the rating dimensions for each participant (five clusters), and (2) the ratings of the random-phase images (four clusters). To find the optimal number of clusters within each approach, we considered the elbow criterion, the silhouette criterion, and the gap criterion. The clearest results were obtained for the elbow criterion while the other criteria yielded ambiguous results. In addition, the number of clusters was chosen so that the number of participants in any cluster exceeded three participants.

## Results

In the present study, we used a convolutional neural network (CNN) to create novel artworks by transferring the artistic style of 25 abstract paintings onto random-phase images with different Fourier spectral properties (see Materials and methods section; Figure 1). In the following sections, we will address the following questions. (1) How do the objective statistical image properties (SIPs) transfer from the input images (original paintings and random-phase images) onto the output (style-transferred) images? (2) How do the subjective ratings of the participants transfer from the input images onto the style-transferred images for the three aesthetic dimensions (Pleasing, Harmonious, and Interesting)? As a special case, we will study the relation between the aesthetic ratings and the initial Fourier power spectra, on which the computer-generated abstract images are based, also for subgroups of participants. In addition, we will study the correlations between the three rating dimensions both across and within participants. (3) Which of the SIPs can predict the aesthetic ratings and are there any differences between subgroups of participants? (4) How do the predictive SIPs in our dataset relate to the image properties of the JenAesthetics dataset of traditional Western paintings and how does this relation predict aesthetic ratings?

### Statistical image properties transfer from the input images onto the style-transferred images

**FIGURE 4:**
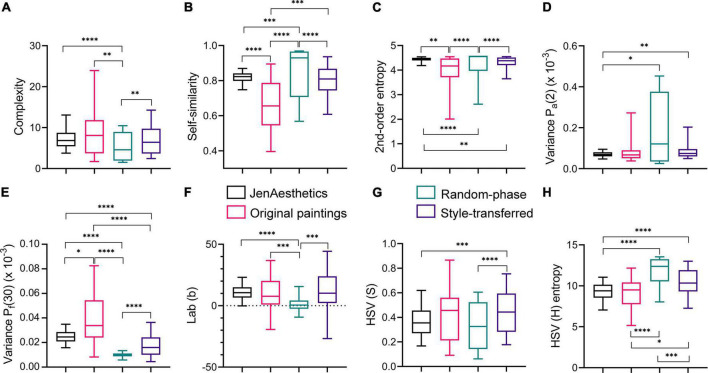
Statistical image properties (SIPs) of the four image categories. The panels **(A–H)** show box plots of the values of all eight SIPs, respectively, as indicated on the y-axis of the plots. In each plot, data are shown for the JenAesthetics dataset of 1629 traditional Western paintings (*black*), the 25 original abstract paintings (*red*), the 150 random-phase images (*green*), and the 150 style-transferred images (*purple*). The boxes encompass the median (*horizontal line*) and represent the 25 – 75 percentiles. The whiskers indicate the 5 – 95 percentiles. Significance levels for the differences between the pairs of image categories are indicated at the top or at the bottom of the panels. Multiplicity-adjusted significance levels are *p < 0.05; ^**^p < 0.01; ^***^p < 0.001; ^****^p < 0.0001.

First, we investigated whether there are differences in the SIPs’ median values between image categories. Figure 4 shows box plots of the eight selected SIPs for the 25 original abstract paintings, the 150 random-phase images and the 150 style-transferred images. For comparison, we show results for the JenAesthetics dataset of traditional Western paintings. As demonstrated before (Redies and Brachmann, 2017; Mather, 2018), the SIPs of the abstract artworks overlap extensively with those of traditional artworks. However, the values for the original abstract art scatter more widely and the median values differ significantly from traditional artworks for three variables (Self-similarity, 2^nd^-order entropy and Variance P_*f*_[30]). As a control, we contrasted the original abstract paintings to a set of 572 abstract artworks from the study by Redies and Brachmann (2017). None of the variables, except for HSV (S), *p* = 0.041, differed significantly, suggesting that the 25 original paintings were representative of a larger body of abstract paintings (data not shown).

As for the random-phase images, all eight SIPs differ significantly from those of the traditional paintings (except for HSV [S]) and the original paintings (except for Variance P_*a*_[2] and HSV [S]), respectively (Figure 4). These objective differences are in accordance with the unique perceptual appearance of the random-phase images (Figures 1D,F,H).

The style-transferred images differ from JenAesthetics paintings in five SIPs (2^nd^-order entropy, Variance P_a_[2], Variance P_f_[30], HSV [S] and HSV [H] entropy) and from the original paintings in three SIPs (Self-similarity, Variance P_f_[30], and HSV [H] entropy). They differ from the random-phase images in all image properties, except for Variance P_a_(2). We thus conclude that the style-transferred images are more similar to the original paintings than to the random-phase images, although both types of images were used in their creation.

Second, the similarity of the input and output images of NST was assessed by correlating the SIPs of the style-transferred images with both the original paintings and the random-phase images. Results are shown in Table 2. All SIPs correlate strongly between the style-transferred images and the original paintings (ρ range: 0.60 – 0.95), with highest ρ values for the three color features. By contrast, only Self-similarity and Variance P_f_(30) showed significant correlations between the style-transferred images and the random-phase images (ρ = 0.61 and 0.36, respectively).

**TABLE 2:** Spearman’s coefficients (ρ) for the correlation between the eight SIPs for all style-transferred images and original paintings as well as the random-phase images, respectively.

SIPs	Original paintings *vs.* style-transferred images	Random-phase images *vs.* style-transferred images
Self-similarity	0.60***	0.61***
Complexity	0.89***	0.06 (n.s.)
2^nd^-order entropy	0.87***	0.089 (n.s.)
Variance P_*a*_(2)	0.80***	–0.044 (n.s.)
Variance P_*f*_(30)	0.72***	0.36***
Lab (b)	0.95***	0.027 (n.s.)
HSV (S)	0.94***	0.013 (n.s.)
HSV (H) entropy	0.87***	0.040 (n.s.)

Significance levels are ***p < 0.001. n.s., not significant.

Third, we took a closer look at the Fourier spectral slope as the random-phase images were produced based on this measure. For the random-phase images, the set (intended) slopes and measured slopes correspond well to each other (Supplementary Figure 2A). This result validates our method of producing the colored random-phase images. Supplementary Figure 2B illustrates that the slope did not translate from the random-phase images to the style-transferred images. For the set slopes of the random-phase images, the slopes measured for the style-transferred images range from –3.3 to –1.8 (median –2.72; 95% CI: –2.73 to –2.71). This range is in fact similar to the range of the 25 abstract paintings in the present study (median: –2.64, 95% CI: –3.11 to –2.49).

### Aesthetic responses transfer from the input images onto the style-transferred images

**FIGURE 5:**
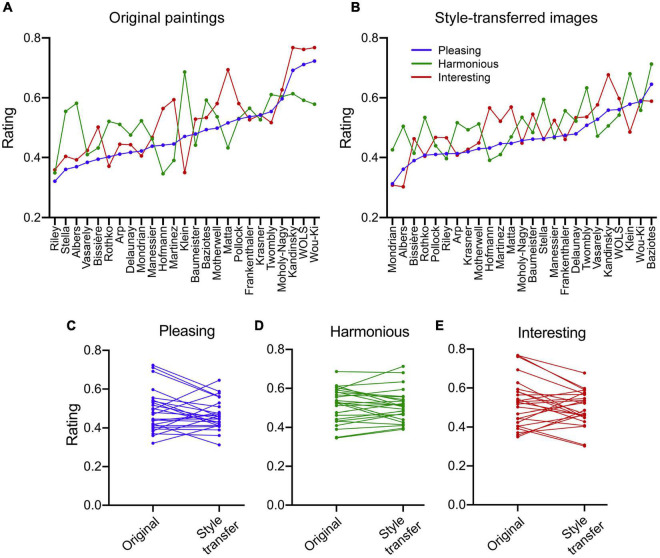
Mean rating responses of participants for the original paintings **(A)** and for their style-transferred counterparts **(B)**. Ratings are shown for Pleasing (*blue*), Harmonious (*green*) and Interesting (*red*). In both panels, individual artists are ordered from left to right in a sequence of ascending Pleasing responses. Spearman’s coefficients (ρ) for the correlations are listed in Table 3. **(C–E)** Same data as shown in **(A,B)** but plotted in slope graphs, separately for the different rating dimensions. Each line connects the mean rating responses for an original painting of one artist and for its style-transferred counterpart.

**FIGURE 6:**
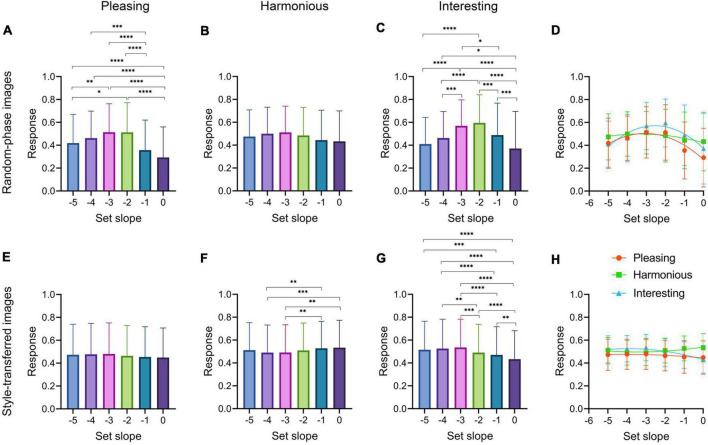
Rating responses for set slope values of the random-phase images. The boxplots show mean responses (y-axis) by all 40 participants for different set Fourier spectral slopes (-5 to 0; x-axis) of the random-phase images **(A–C)** and the style-transferred images **(E–G)**. The whiskers represent the 5 – 95% confidence intervals. The rating dimensions are indicated on the top of the panels [**(A,E)** Pleasing; **(B,F)** Harmonious; and **(C,G)** Interesting]. Multiplicity-adjusted significance levels of pairwise comparisons are indicated by the asterisks (*p < 0.05; ^**^p < 0.01; ^***^p < 0.001; ^****^p < 0.0001). Panels **(D)** and **(H)** show least-square fittings of second-order polynomial (quadratic) functions to the data from the previous three panels (*orange*, Pleasing; *green*, Harmonious; and *blue*, Interesting).

Each of the three image categories elicits a wide range of aesthetic ratings in the beholder (Figures 5, 6). In the following sections, we will describe how the subjective ratings transfer from the input images (original paintings and random-phase images) to the output (style-transferred) images.

Figure 5 shows the mean ratings per artist for the original paintings (Figure 5A) and for the style-transferred images (Figure 5B), respectively. Artworks are sorted from left to right according to the Pleasing ratings. The sequence of the artists from low to high ratings is roughly similar for the two image categories (Figures 5C–E). We thus correlated the ratings of the original paintings and the style-transferred images and found that the mean responses per artist correlate for all three rating dimensions, but to different degrees (Spearman’s ρ range: 0.48 – 0.80; Table 3). In other words, if participants rated particular original paintings more highly, they tended to do so also for their style-transferred derivatives. Unlike the ratings for the original paintings, the ratings of the random-phase images did not correlate significantly with those of the style-transferred images (Table 3).

**TABLE 3:** Spearman’s coefficients (ρ) for the correlations between the three rating dimensions for all style-transferred images and original paintings as well as the random-phase images, respectively.

Rating dimensions	Original paintings *vs.* style-transferred images	Random-phase images *vs.* style-transferred images
Pleasing	0.48*	0.03 (n.s.)
Harmonious	0.80***	0.05 (n.s.)
Interesting	0.52**	0.09 (n.s.)

Significance levels are *p < 0.05; **p < 0.01; ***p < 0.001. n.s., not significant.

#### Random-phase images with different set slope values

To create the style-transferred abstract images, we used random-phase images that possessed slopes of the Fourier power spectrum ranging from –5 to 0. We thus asked whether the rating responses for the different set slope values transferred from the random-phase images onto the style-transferred images. Results are plotted as a function of the Fourier slope in Figure 6. We will first consider the ratings for the random-phase images, followed by the style-transferred images. Note that on a descriptive level, the style transfer did not translate the original slopes from the random-phase images to the output images, as described above (Supplementary Figure 2).

For the random-phase images, rating responses for Pleasing and Interesting follow an inverted u-shape with highest responses for slopes of –2 and –3 (Figures 6A,C). Differences are not significant for Harmonious ratings (Figure 6B). These results were confirmed by least-square fitting of 2^nd^-order polynomial (quadratic) functions (Figure 6D). Our findings thus extend results by Spehar et al. (2016) for grayscale random-phase images into the color domain.

For the corresponding style-transferred images, participants tended to rate the style-transferred images as more Interesting if they were derived from random-phase images with set slope values of less than –2, with a maximum at a set slope value of –3 (Figure 6G, *blue* in Figure 6H). However, the differences are less pronounced than those of the random-phase images. Interestingly, there is a weak inverse relation between set slope values and responses for Harmonious with lower responses for set slope values of –5 to –2 (Figure 6F, *green* in Figure 6H). For Pleasing, no differences in the ratings were obtained for different set slope values (Figure 6E, *orange* in Figure 6H). Taken together, our data suggest that the transfer of ratings from the random-phase images onto the style-transferred images is much less effective than from the original paintings.

Previous results by other researchers (Bies et al., 2016; Güclütürk et al., 2016; Spehar et al., 2016) revealed that individual participants favor different degrees of complexity in random-phase patterns. We thus asked whether groups of participants differed in their taste also for the colored versions of the random-phase images. Hence, we clustered participants according to the mean responses of each participant per set slope for all three rating dimensions. About half of the participants (Clusters 1 and 2) exhibit an inverted u-shaped response curve for all three rating dimensions. Linearly decreasing or increasing slope values were found for the remaining clusters (for detailed results, see Supplementary Figure 3).

#### Inter-rating correlations

Table 4 lists correlations between the rating dimensions for all three image categories across all participants. The lowest correlations are observed between Harmonious and Interesting while both dimensions correlate more highly with Pleasing. Figures 5A,B illustrates that ratings for Harmonious and Interesting vary widely for many artists.

**TABLE 4:** Spearman’s coefficients (ρ) for the correlations between the different rating dimensions (Pleasing, Harmonious, and Interesting) for all participants.

	Pleasing *vs.* Harmonious	Pleasing *vs.* Interesting	Harmonious *vs.* Interesting
Original paintings (*n* = 25)	0.54	0.73	0.36
Random-phase images (*n* = 150)	0.56	0.56	0.29
Style-transferred images (*n* = 150)	0.48	0.53	0.25

All significance levels, *p* < 0.0001.

Despite these general tendencies, we observed marked differences between participants in the correlations between the rating dimensions (data not shown). Therefore, we calculated the inter-rating correlations also within participants and clustered participants according to these correlations. Results for the five clusters obtained (Table 5) indicate that the overlap of Pleasing with Harmonious and Interesting, respectively, is about equally strong for most participants. By contrast, Harmonious and Interesting correlate less strongly with each other (see also Figures 5A,B) and some participants even showed anticorrelated response tendencies. However, these results should be considered to be preliminary because the number of participants in the different clusters is very small (Dalmaijer et al., 2022).

**TABLE 5:** Average Spearman’s coefficients (ρ) for the correlations between the different rating dimensions (Pleasing, Harmonious, and Interesting) for the five groups of participants that were clustered on the basis of the inter-rating correlations.

	Pleasing *vs.* Harmonious	*p*	Pleasing *vs.* Interesting	*p*	Harmonious *vs.* Interesting	*p*
Cluster 1 (*n* = 14)	0.55	<0.0001	0.64	<0.0001	0.47	<0.0001
Cluster 2 (*n* = 13)	0.44	<0.001	0.39	<0.01	0.31	<0.01
Cluster 3 (*n* = 4)	0.64	<0.0001	0.29	<0.05	0.01	0.51 (n.s.)
Cluster 4 (*n* = 5)	0.21	<0.05	0.56	<0.0001	0.04	0.23 (n.s.)
Cluster 5 (*n* = 4)	0.04	0.37 (n.s.)	0.32	<0.05	–0.34	<0.001
All (*n* = 40)	0.48	<0.0001	0.53	<0.0001	0.25	<0.0001

n.s., not significant.

### Statistical image properties explain aesthetic ratings

**FIGURE 7:**
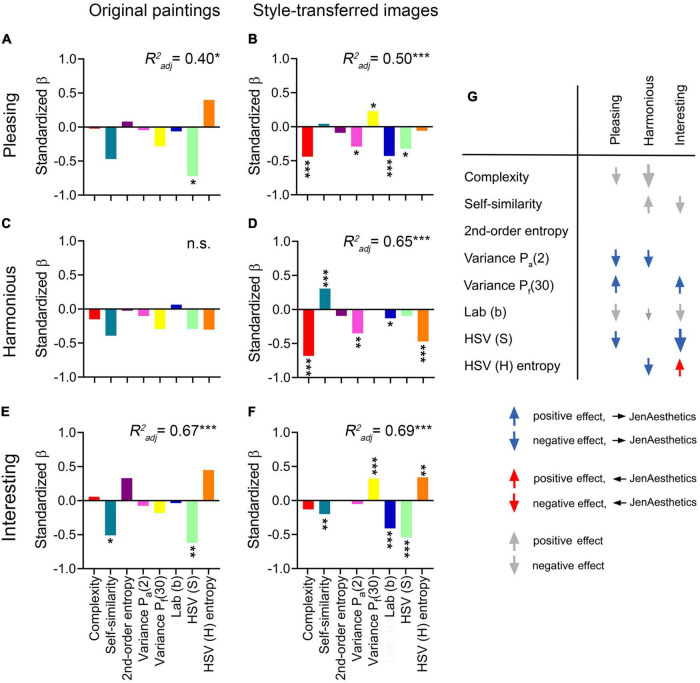
Standardized β (β*) values for the influence of the statistical image properties (SIPs) on the rating responses. Data are shown for original artworks **(A,C,E)** and style-transferred images **(B,D,F)**. The three rating dimensions are Pleasing **(A,B)**, Harmonious **(C,D)** and Interesting **(E,F)**. The explained variance (*R^2^_adj_*) of the respective model is indicated on top of each panel. Asterisks indicate β* values of variables that had a significant effect on the ratings when the other variables were controlled for; the respective significance levels are *p < 0.05; **p < 0.01; ***p < 0.001. n.s., not significant. **(G)** Influence of the SIPs on the rating responses, in relation to the JenAesthetics dataset. This overview summarizes results for the ratings of the style-transferred images for all participants **(A–F)**. The influence of the eight independent variables (SIPs) on the ratings (Pleasing, Harmonious, and Interesting) is represented by arrows, which are shown only for those variables that had a significant effect on the ratings when the other variables were controlled for [marked by asterisks in **(B,D,F)** and Supplementary Table 2]. The size of the arrows indicates the strength of the relation [small arrows, | β*| < 0.2 (weak effect); medium-sized arrows, 0.2 ≤ | β*| < 0.5 (moderate effect); and large arrows, | β*| ≥ 0.5 (strong effect)]. The direction indicates the sign of the relation (upward, positive relation; and downward, negative relation). The colors indicate the changes relative to the results for the JenAesthetics data set (Figure 4). Blue arrows indicate higher ratings if the SIPs are closer to the mean SIPs of the JenAesthetics data set. Red arrows indicate higher ratings if the SIPs are more distant from the mean SIPs of the JenAesthetics data set. Gray arrows indicate no significant differences of the SIPs between the style-transferred images and the JenAesthetics data set.

To determine how well the SIPs explain the aesthetic responses along the three rating dimensions, we performed a multiple linear regression analysis with a model that comprised the eight independent variables (SIPs) selected for our analysis (see Materials and methods section). In the following two sections, we will describe how each variable predicts the ratings of the style-transferred images and compare the results to the original paintings (Figure 7). As described in the Statistical methods section, we refer to the β* coefficients as weak, moderate, and strong effects, respectively. Because the random-phase images display a rather unique image structure and differ in their image properties from both the original paintings and the style-transferred images, we will not consider them in the analysis of how SIPs explain the aesthetic ratings.

#### Style-transferred images

Figure 7 and Supplementary Table 2 list the explained variance for each model (*R^2^_adj_*) and the β* coefficient for each SIP. Overall, the SIPs predict a relatively large part of the observed variance in the ratings. Except for 2^nd^-order entropy, all other SIPs predict the responses to the style-transferred images for at least two of the rating dimensions (weak to strong effects, Figures 7B,D,F). Moreover, the direction of the β* coefficients is the same for the three rating dimensions for most SIPs. Positive β* values are obtained for Variance P_f_(30) (Pleasing and Interesting), and negative β* values for Complexity and Variance P_a_(2) (Pleasing and Harmonious), and Lab (b) and HSV (S) (Pleasing and Interesting). Only Self-similarity and HSV (H) entropy show opposite directions for Harmonious and Interesting, respectively. Lower levels of Self-similarity are perceived as more Interesting (Figure 7F) whereas higher levels of Self-similarity are rated as more Harmonious (Figure 7D) in the style-transferred images. The opposite tendency is seen for HSV (H) entropy. Here, higher values for Variance P_f_(30) are perceived to be more Pleasing and Interesting (Figures 7B,F).

#### Original paintings

Compared to the style-transferred images, significant predictors (asterisks in Figures 7A,C,E) are less numerous for the original paintings. This result is expected because the SIPs were selected based on the style-transferred images (see Materials and methods section). Moreover, the size of the sample (25 original paintings) is exceedingly small for statistical analyses, which must therefore be considered preliminary. Nonetheless, the data suggests that participants prefer original paintings with lower values for the variables Self-similarity and HSV (S) for all three rating dimensions. For Self-similarity, preferred images are rated more highly if values are more different from the mean values of all other image categories (Figures 4B,7A,C,E). For increasing values of HSV (H) entropy, ratings increase for Pleasing and Interesting, while the opposite relation is seen for Harmonious (Figures 7A,C,E). For the sake of completeness, results for random-phase images are listed in Supplementary Table 2.

#### Clustering participants according to inter-rating correlations

As described above, participants were clustered according to the correlations of rating responses along the three rating dimensions (Table 5). Supplementary Figure 4 and Supplementary Table 3 show the results of the multiple linear regression model for the five clusters. All models are significant with explained variances ranging from 0.19 to 0.78. The relation between the inter-rating correlations and the preferences for particular SIPs can be described as follows. Clusters 1 and 2 show about equally strong correlations between all three rating dimensions. Correspondingly, participants preferred images with similar SIPs for all three rating dimensions. Stronger inter-rating correlations in Cluster 1 than in Cluster 2 correspond to more predictive power of the SIPs in Cluster 1. Second, in Cluster 3, the stronger correlation between ratings of Pleasing and Harmonious is mirrored by a similar pattern of β* values for the two rating dimensions. Third, Cluster 4 lacks a correlation between the ratings of Harmonious and Interesting. Accordingly, the SIPs that are associated with these ratings differ. Fourth, there is a negative correlation between ratings of Harmonious and Interesting in Cluster 5, which is also reflected in opposite signs of the β* values. Again, these preliminary results await confirmation by clustering studies with more participants.

### Higher aesthetic ratings for statistical image properties that resemble traditional Western paintings

We next studied the rating responses of the style-transferred images and the relation of their SIPs and those of the JenAesthetics dataset of traditional Western paintings. We speculated that style-transferred images are rated more highly if their SIPs are closer to those of the JenAesthetics dataset (see Introduction section). To address this hypothesis, we examined the five variables that differed between the style-transferred images and the JenAesthetics images (2^nd^-order entropy, Variance P_a_[2], Variance P_f_[30], HSV [S], and HSV [H] entropy; Figures 4C–E,G,H). For most of these variables, responses are higher if the values of the SIPs are closer to those of the JenAesthetics images (blue arrows in Figure 7G). In other words, if the median SIP value of the JenAesthetics dataset is lower than that of the style-transferred images, β* values are negative. Consequently, the style-transferred images with smaller SIP values are rated more highly (as an example, see Pleasing and Interesting ratings for HSV [S]; Figure 7G). If the median SIP value of the JenAesthetics dataset is higher than that of the style-transferred images, the inverse applies. For HSV (H) entropy only, Harmonious and Interesting ratings show opposite tendencies in comparison to the JenAesthetics dataset (blue arrow and red arrow in Figure 7G, respectively). For 2^nd^-order entropy, the effect on the ratings is not significant in the model (Figure 7G) although the mean values for style-transferred images and the JenAesthetics images differ (Figure 4C).

**FIGURE 8:**
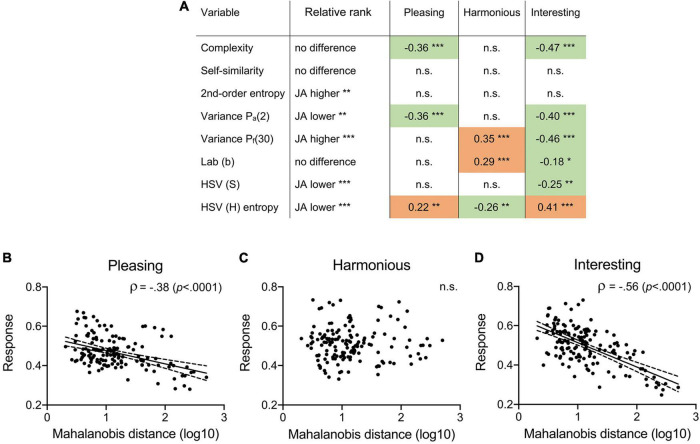
Influence of the SIPs on the rating responses to the style-transferred images in relation to the median SIP values of the JenAesthetics (JA) dataset. **(A)** Spearman coefficients ρ for the correlation between the rating responses and the Euclidean distance between each individual SIP and the median SIP of the JenAesthetics dataset, respectively. Negative correlations (*green*) imply that the ratings are higher if the SIPs are closer to the JenAesthetic dataset. The inverse holds for positive correlations (*orange*). The second column lists the rank of the style-transferred images relative to the JA dataset. **(B–D)** Responses for each rating dimension are plotted as a function of the Mahalanobis distance in the 5d space spanned by the five SIPs that differ significantly between the style-transferred images and traditional Western artworks (Figure 4). Each dot represents one style-transferred image. For the linear regression, the solid line represents the fitted line and the dashed lines its 95% confidence interval. Spearman’s coefficients of correlation ρ are given in **(A)** and **(C)** with their respective significance levels. For **(A–D)**, significance levels are *p < 0.05; **p < 0.01; ***p < 0.001. n.s., not significant.

For each SIP, we then correlated the rating responses with the Euclidean distance between the style-transferred images and the median of the JenAesthetics dataset (Figure 8A). We find strongest negative correlations for Interesting ratings which suggests that style-transferred images are rated as more interesting, if their SIPs approach those of the JenAesthetics dataset (green shadings in Figure 8A). Similar, yet less consistent results can be found for Pleasing ratings. An interesting exception is HSV (H) entropy where images are rated as more Pleasing and Interesting, the more distant they are from the JenAesthetics dataset, and more Harmonious, the closer they are.

To substantiate the above result, we calculated the Mahalanobis distance of each style-transferred image to the median of the JenAesthetics dataset in the multidimensional space spanned by the five SIPs. We correlated the distances with the aesthetic ratings. Results in Figures 8B–D suggest that style-transferred images, which are located closer to the JenAesthetics dataset in this space, are rated as more highly for Pleasing and Interesting; no such correlation is found for Harmonious ratings.

## Discussion

We investigated how neural style transfer (NST; Gatys et al., 2015; Kolkin et al., 2019) can be used to generate abstract images that display a wide range of statistical image properties. With these images, we pursued four aims to better understand the style transfer process. (1) We compared the objective properties (SIPs) and (2) the ratings of the input images (original artworks and random-phase images) with the output images (style-transferred images). (3) We asked which SIPs predict aesthetic ratings by human beholders in the style-transferred images and (4) how these SIPs and their predictive value for aesthetic ratings relate to those of a large set of traditional Western paintings (JenAesthetics dataset).

To describe the objective structure of the images, we selected a set of eight statistical image properties (SIPs) that have been related previously to artistic style and aesthetic perception. The selected SIPs cover different aspects of formal image structure and composition. They reflect the density and distribution of oriented luminance and color gradients (Complexity, Self-similarity, 2^nd^-order entropy), richness and variability of low-level CNN filter responses (Variance P_a_[2] and P_f_[30]) and color features (Lab [b], HSV [S], and HSV [H] entropy). For the style-transferred images, the eight SIPs assumed a wide range of values (Figure 4) and showed relatively weak correlations between each other (Figure 3).

Importantly, the eight SIPs were strong predictors of the aesthetic rating responses to the style-transferred images (Figures 7,8 and Supplementary Tables 2,3). The explained variances *R^2^_adj_* for models with the eight SIPs are about as high as the *R^2^_adj_* values for models with the first eight principal components of all 29 variables that were considered initially (Supplementary Table 2; see Materials and methods section). Thus, the reduction from 29 to 8 variables did not decrease the explanatory power of the reduced model substantially.

We can only speculate about the origins of the remaining variance, which is not covered by the SIPs. Besides higher-order visual features, possible sources of variance include environmental and genetic factors (Bignardi et al., 2020; for a review, see Chamberlain, 2022), as also found for the evaluation of face attractiveness (Germine et al., 2015). Personality factors also predict aesthetic ratings (Chamorro-Premuzic, 2009). For instance, they explain a large proportion of the variance associated with aesthetic chills in response to art (Silvia and Nusbaum, 2011; Bignardi et al., 2022). A comprehensive model on how these diverse factors interact remains elusive at present.

### Transfer of statistical image properties during neural style transfer

Our aim was to quantify how the SIPs changed during their transfer from original artworks onto random-phase images. We found that the style-transferred images differ from original paintings in three SIPs and from random-phase images in seven SIPs. In other words, the style-transferred images resemble original abstract artworks more closely in their image properties than they resemble the random-phase images. The correlation analyses (Table 2) quantify the transfer effects and provide evidence that what was originally termed the “style image” (Gatys et al., 2015) determines the formal features, i.e., the SIPs, whereas the formal features of the “content image” (Gatys et al., 2015) get largely lost in the process of NST. This result suggests that style, as defined in NST (Gatys et al., 2015), can be represented, at least in part, by the eight SIPs in our study. In particular, the transfer of color features seems to work particularly well both subjectively (Figure 1) and objectively, as indicated by high correlations between color values and ratings (Tables 2,3).

As an example, the Fourier slope, which was set to fixed values of –5 to 0 in the random-phase images, transforms to a relatively narrow range of values between –3 and –2 in the style-transferred images (Supplementary Figure 2). The 25 abstract paintings in the present study (–3.34 to –1.59; median: –2.64) also fall within this range. This range of values is close to the Fourier slope of natural scenes and other visual artworks (Aks and Sprott, 1996; Graham and Field, 2007; Redies et al., 2007), which human beholders generally prefer (Graham and Redies, 2010).

The range of SIPs of the style-transferred images shows considerable overlap with human-made artworks (Figure 4). The variance of the individual SIPs of the style-transferred images is generally higher than that found in traditional Western paintings (JenAesthetics dataset; Figure 4). A large range of variation of SIPs has also been described for abstract art (Redies and Brachmann, 2017) and modern art (Mather, 2018). The SIPs of the style-transferred images thus represent a wide range of values that cover also those of traditional art and abstract/modern art.

### Transfer of aesthetic ratings during neural style transfer

Our results revealed that mean rating responses for the original abstract paintings correlate positively with their style-transferred derivatives. This correlation was particularly high for Harmonious and lower for Pleasing and Interesting. A similar correlation was not found between the style-transferred images and the random-phase images. These findings confirm our hypothesis that not only the visual appearance (i.e., image style, as manifested by the SIPs) but also the aesthetic preferences are largely derived from the original abstract paintings rather than from the random-phase images during NST. Together, our results suggest that we successfully created a novel type of image that shared objective and subjective properties with original abstract artworks, and were not just copies of the input images. A similar notion was put forward originally by Gatys et al. (2015).

Several studies described that participants share a prejudice against computer-generated art. For example, Chamberlain et al. (2018) studied aesthetic responses to artworks created either by humans or computers. While participants were able to readily distinguish between these two categories of images, they were prejudiced against computer-generated artworks (see also Ragot et al., 2020). This prejudice, however, is partially overcome by alerting participants to human-like characteristics of the computer algorithms (Chamberlain et al., 2018) or by attaching randomly generated pseudo-profound “bullshit” titles to computer-generated paintings (Turpin et al., 2019). Using generative adversarial networks (GANs), Elgammal et al. (2017) demonstrated that computers can create artworks, which participants cannot distinguish from art generated by contemporary artists. In the present study, we show that different sets of SIPs predicted the aesthetic ratings of original paintings and style-transferred images (Figure 7). Because we did not ask our participants to discriminate between computer-generated and man-made artworks, we do not know whether the computer-generated images were recognized as such or were rated in a biased way.

As a side finding, we studied the preferences for the Fourier spectral slopes of colored random-phase images, following a previous study by Spehar et al. (2016) for grayscale random-phase images. The authors discovered individual differences between groups of participants. About half of their participants show the well-known inverted u-shaped response curve with increasing slope values (Wundt, 1874), about 20% each show an increasing or decreasing curve, respectively. Similar groups with ascending and descending preference curves were observed by Bies et al. (2016) and Güclütürk et al. (2016). Our results extend these findings to colored versions of the same type of stimuli. When participants were clustered according to their preferences for the slope of random-phase images in our study, about half of the participants retained their inverted u-shaped response curves (Clusters 1 and 2 in Supplementary Figures 3A,B), while response rates for other clusters increased and/or decreased with increasing slope values (Supplementary Figures 3C,D).

A selective preference for random-phase patterns with different slopes can also be found for thresholded and edges-only derivatives of these patterns (Spehar et al., 2016). We therefore asked whether the slope preferences also transfer to the style-transferred images during NST. The objective slopes themselves did not transfer onto the style-transferred images (Supplementary Figure 2). For the aesthetic ratings, an inverted u-shaped curve was found only for Interesting ratings (Figure 6G), but it is much less pronounced than for the random-phase images (Figures 6D,H). The original set slopes of the random-phase images only have minor effects on the ratings of Harmonious and Interesting (Figures 6F,G). These findings suggest that the original abstract paintings mediate style transfer predominantly.

### High predictability of average aesthetic ratings

The above results suggest that the artificially generated images are well-suited to study the effect of SIPs on aesthetic ratings of artworks. Indeed, a large part of the variance in the ratings of the style-transferred images can be explained by eight SIPs (*R^2^_adj_* ranging from 0.50 to 0.69; Figures 7B,D,F and Supplementary Table 2). We find that average ratings for all three aesthetic dimensions can be explained by the same set of eight SIPs (Figures 7B,D,F). This finding is unlike results from previous studies. Vessel and Rubin (2010) found stronger shared taste for real-world images as opposed to abstract images. They proposed that common semantic interpretations of real-world images lead to a more uniform experience across observers whereas reactions to abstract images are more idiosyncratic. This conclusion is in line with results by Leder et al. (2016) who found that the proportion of shared taste was much higher for faces than for abstract artworks. The authors suggested that participants possibly do not agree on a concept of shared taste for abstract art (Leder et al., 2016).

The relatively high explanatory power of image style, as represented by the SIPs, on aesthetic ratings in our study might be explained by the rather homogenous appearance of the style-transferred images, as compared to the stimuli studied by Vessel and Rubin (2010). Consequently, differences in the aesthetic ratings might be more closely associated with specific SIPs in our study, as previously observed by Stanischewski et al. (2020) in simple line patterns. Alternatively, the high explanatory power may be due to the fact that the original abstract images and the style-transferred images were rated in separate blocks; participants might have used multiple non-interacting rating scales that adapt well to the different types of stimuli (Vessel and Rubin, 2010). Last but not least, our stimuli are abstract, so that depicted content cannot affect ratings of formal image structure.

### Statistical image properties closer to traditional paintings predict higher aesthetic ratings of style-transferred images

We found that style-transferred images that are more similar in their SIPs to traditional Western paintings (JenAesthetics dataset), are rated as more Pleasing and Interesting (Figures 4, 8). For Harmonious, the ratings and the Mahalanobis distance did not correlate significantly.

Previous findings indicate that traditional paintings cluster at particular positions (here called “sweet spots”) in spaces that are spanned by specific SIPs (Redies et al., 2012; Braun et al., 2013; Brachmann et al., 2017; Redies and Brachmann, 2017). Interestingly, the sweet spots for Western, Islamic, and Chinese paintings were found to overlap to a large degree (Brachmann and Redies, 2017; Redies and Brachmann, 2017). These and other results (reviewed in Che et al., 2018; Nakauchi et al., 2022) are compatible with the notion that traditional artworks – as opposed to some types of modern art (Redies, 2014; Redies and Brachmann, 2017) – exhibit a restricted set of visual cues that are universally appreciated within and across cultures. This notion of universal beauty as an intrinsic perceptual property of artworks has a long tradition in art theory. For example, Bell (1914) claimed that visual artworks possess a “significant form,” which can elicit an aesthetic experience that is universal amongst humans and is unrelated to the cultural context or the displayed content of artworks. In the present study, we observed that formal properties of images (i.e., the SIPs) correlate with higher aesthetic ratings when they are closer to the sweet spot, where traditional paintings are represented. However, the existence of a significant form (sweet spot) has been contested by some contemporary researchers (for examples, see Conway and Rehding, 2013; Zeki, 2013).

### Differences between rating dimensions

The rating dimensions used in the present experiment reflect three components of aesthetic experience (Berlyne, 1974; Marković, 2012): hedonic tone (Pleasing), regularity (Harmonious), and arousal (Interesting). In all three image categories, Harmonious and Interesting represent relatively independent rating dimensions (Spearman coefficients ρ range: 0.25 to 0.36; Table 4) whereas Pleasing/Harmonious and Pleasing/Interesting correlate to a higher degree (ρ range: 0.48 to 0.73). These findings, particularly the low correlation between Harmonious and Interesting, are substantiated by the following results. First, the correlation of the two rating dimensions with the SIPs has an opposite direction for Self-similarity and HSV (H) entropy (Figure 7). Second, mean ratings for individual artists tend to assume diverging values for Harmonious and Interesting (Figures 5A,B). Third, different groups of participants seem to interpret the rating dimensions differently, as reflected by the clusters based on the inter-rating correlations (Table 5). Here, the mean coefficients for the correlations between Harmonious and Interesting range from 0.47 (Cluster 1) to –0.34 (Cluster 5). Fourth, correlations between the ratings of Harmonious/Interesting and the distance to the JenAesthetics dataset tend to assume opposite signs (Figure 8A).

Our findings are consistent with results by Schwabe et al. (2018) who examined the perception of abstract artworks with another method that largely prevents cognitive processing, i.e., ultrashort exposure times (gist perception). The authors found that structure-related terms such as Harmonious are more stable and consistent under these viewing conditions than cognition-related ratings such as Interesting, while ratings of Pleasing are inconsistent. In contrast to harmony, which has been less well investigated, several studies have addressed the role of pleasure and interest in aesthetic judgments. Berlyne (1974) described that interestingness and pleasingness vary with the same independent variables (for example, complexity and novelty) but the exact nature of relations differs. Silvia (2005) interpreted interest as an emotion that consists of appraisals of novelty and coping potential. Fayn et al. (2015) found that the personality trait of Openness was predictive of greater interest and pleasure in response to visual art. Last but not least, in their fluency-based hierarchical model of aesthetic liking, Graf and Landwehr (2015) invoked pleasure and interest as the major outcomes of stimulus-driven processing and perceiver-driven processing, respectively.

Groups of participants also differ in their aesthetic taste when clusters are formed according to inter-rating correlations (Supplementary Figure 4). Such differences in aesthetic taste have been linked to personality traits. For example, the trait Openness predicts preference for abstract art over other art styles (Furnham and Walker, 2001) and over Renaissance art, respectively (Pelowski et al., 2017). Lyssenko et al. (2016) demonstrated that, within abstract art, preferences for particular SIPs correlated with individual personality traits. Besides complexity, preference for other SIPs is shared by groups of participants, for example, for color and self-similarity (Mallon et al., 2014) as well as for curvature (Cotter et al., 2017).

### Methodological limitations

Our study has the following limitations. (1) We devised our stimuli with the intention that they do not show any figurative content. On the one hand, this lack of figurative cues is advantageous for studying perceptual aspects of aesthetic judgments because figurative elements do not confound rating responses. On the other hand, our approach cannot take interactions between perception of aesthetic form and processing of image content and context into account (Locher et al., 1999; Leder et al., 2004; Estrada-Gonzalez et al., 2020). Such interactions can occur for ordinary aesthetic experiences outside the laboratory setting (Specker et al., 2017). Whether our conclusions also hold for more “natural” aesthetic experiences thus remains to be studied. (2) The vast majority of the 40 participants in the rating experiments were students of medicine and art history in Jena, Germany. They rated style-transferred images that represent a relatively uniform set of abstract images. In how far their aesthetic ratings are representative also for other (larger) groups of beholders, other cultural backgrounds, or other artistic styles is unclear. (3) The sample size of original paintings (*n* = 25) analyzed in the present study is exceedingly small and can thus not deliver robust statistical results. (4) In order to avoid problems in our statistical analyses, such as overfitting and multicollinearity, we reduced the number of independent variables to a relatively small subset of eight SIPs. It remains to be studied whether this subset can also predict aesthetic ratings of other datasets of artworks. (5) Ratings for the three types of images studied cannot be compared directly in absolute terms because they were tested in separate blocks. Under these conditions, participants are likely to use multiple non-interacting scales for the different types of stimuli (Vessel and Rubin, 2010). (6) By presenting the original paintings as the last (third) block, we cannot exclude the possibility that the ratings are affected by the first two blocks (Figure 2). (7) Last but not least, the present findings are based on the NST method by Kolkin et al. (2019) and it is unclear whether they generalize to other NST methods.

## Conclusion

Our results suggest that NST can be used to create novel abstract images that possess statistical image properties similar to those of the original artworks. Moreover, the participants’ preferences partly transfer onto the novel images. The novel images are rated higher if their SIPs assume values closer to those of the JenAesthetics dataset of traditional Western paintings (“sweet spot”). We were able to explain a large portion of the aesthetic ratings with a representative set of only eight SIPs. We see these results as a successful example of how to use NST-generated images in experimental aesthetics research. Whether the images created in the present study can be considered genuine artworks depends on how we define creativity and art. At present, these terms lack a precise and objectifiable definition. Nevertheless, we agree with other researchers (Lomas, 2018; Hertzmann, 2019; McCormack et al., 2019) that computers provide a highly versatile artistic medium, which can assist artists and serve as an engine for artistic innovation.

## Data availability statement

The raw data supporting the conclusions of this article are available at the website of the Open Science Framework (osf.io/mh74t).

## Ethics statement

The studies involving human participants were reviewed and approved by Ethics Committee of Jena University Hospital (approval no. 2021-2223-Bef). The patients/participants provided their written informed consent to participate in this study.

## Author contributions

HG and CR conceived the experiments, analyzed and visualized the data, and prepared the draft of the manuscript. RB wrote the software and produced the stimuli. HG carried out the rating studies and collected the experimental data. KT provided statistical advice. HG, RB, KT, and CR reviewed and edited the manuscript. All authors read and agreed to the published version of the manuscript.

## Footnotes

1 Kolkin, N. (2020). *STROTSS*. Available at: https://github.com/nkolkin13/STROTSS (Accessed: 16 July 2020).
