# Supplementary material for: Statistical image properties predict aesthetic ratings in abstract paintings created by neural style transfer
Source: Front Neurosci. 2022 Oct 13;16:999720. doi: 10.3389/fnins.2022.999720 (PMC9606769; doi:10.3389/fnins.2022.999720)
Supplement: Supplementary file 1 [file Data_Sheet_1.PDF]

Supplementary Material

1 Description of Statistical Image Properties

1.1 PHOG Complexity, Self-similarity, and Anisotropy

The three SIPs were calculated using the Pyramid Histogram of Oriented Gradients (PHOG) method, which was initially developed for object recognition and image classification in computer vision (Bosch et al., 2007). After converting an image into its representation of luminance and color gradients (called gradient images), histograms of oriented gradients (called HOG descriptors; Dalal and Triggs, 2005) were calculated for pyramidal subdivisions of the gradient image (for a detailed description of the method, see the Appendix in Braun et al., 2013).

Image Complexity was expressed as mean gradient strength for all subdivisions of the gradient image. It overlaps with other SIPs that are known to reflect image Complexity, including edge density, the overall signal strength in Gabor-filtered images and the fractal dimension (for reviews, see Machado et al., 2015; Van Geert and Wagemans, 2020). The degree of Complexity contributes to image preference of a large variety of visual patterns. Different groups of beholders prefer different degrees of Complexity (Bies et al., 2016; Güçlütürk et al., 2016; Spehar et al., 2016).

Self-similarity of an image is calculated by determining how similar the HOG descriptors for the subsections are to those of other subsections at different levels of the pyramid or to the ground level histogram (Bosch et al., 2007; Amirshahi et al., 2012). Higher Self-similarity values indicate that the subsections of an image contain gradients whose orientation histograms are more similar to other subsections of the image. In the present study, we compared histograms of all levels up to level 3 of the pyramid (8×8 grid, resulting in 64 subdivisions at pyramid level 3) with the histogram of the ground image (32 orientation bins). An intermediate to high degree of Self-similarity is associated with traditional paintings and other visually pleasing stimuli (Braun et al., 2013).

The HOG approach can also be used to determine how different the strength of the gradients is across orientations in an image ("anisotropy"). If the strength of all oriented gradients is uniform across orientations, i.e. all orientations are of about equal strength, HOG anisotropy decreases with a lower limit of zero. If particular orientations predominate in an image, e.g. horizontal and vertical orientations in an architectural photograph, anisotropy is high. PHOG anisotropy correlates highly inversely with 2nd-order entropy of edge orientations (Spearman coefficient $\rho = -0.72$; see next paragraph) and was therefore excluded from the final set of 8 variables (Table 1).

1.2 Entropy of Edge Orientations

Summary statistics for the prevalence of luminance edges and their orientations in an image can also be obtained by filtering an image with a set of oriented Gabor filters, which can be viewed as models of simple cell responses in the primary visual cortex. In histograms that cover all orientations at equal intervals, we plotted the strength of the response as a function of filter orientation (Geisler et al., 2001). As a measure of histogram uniformity, Shannon entropy was calculated. Entropy is maximal

if response strength is the same across all orientations. It decreases as particular orientations become more prominent in the histograms. We computed first-order and second-order entropy of edge orientations. First-order entropy refers to global histograms for Gabor responses (oriented edges) that are summarized for the entire image. Second-order entropy reflects pairwise statistics of edge orientations across an image. To obtain this measure, we compared the orientation of all edges to the orientation of all other edges by pairwise comparison in each image (Geisler et al., 2001; Redies et al., 2017). Entropy is maximal if all orientation differences are equally likely to occur, i.e. the orientations of the edge pairs are independent of each other across an image. Entropy is lower in images where particular combinations of orientations abound, for example, edges of parallel or orthogonal orientations. Pairwise statistics of edge orientations have been studied in the context of contour grouping and natural scene statistics (Geisler et al., 2001; Sigman et al., 2001).

In a previous study, we described that 2nd-order edge orientation is relatively high in large subsets of traditional artworks of different cultural provenance (Redies et al., 2017) and in other types of visually pleasing images (Grebenkina et al., 2018). Because 1st-order entropy is generally high in images with high 2nd-order entropy, the two measures correlate strongly. Indeed, the Spearman coefficient ρ for the 150 style-transferred images was 0.84. Therefore, we excluded 1st-order Entropy from the set of SIPs used the final analysis (Table 1).

1.3 Variances of Feature Responses in Convolutional Neural Networks (CNNs)

CNNs can predict the aesthetic quality of images in rating tasks (Lu et al., 2015; Rafegas and Vanrell, 2016; for a review, see Brachmann and Redies, 2017). A draw-back of CNNs is the lack of interpretability of individual feature responses, especially at higher levels. To classify visual artworks, we proposed to study variances of filter responses at lower levels of the AlexNet CNN model, as described in detail by Brachmann and Redies (2017). This method captures the distribution of luminance and color edges (Rafegas and Vanrell, 2016) as well as spatial frequency filters across images and allows to classify traditional artworks with high accuracy. At the same time, the feature responses can be interpreted in terms of low-level neural responses up to cortical area V1.

In the present study, we calculated filter response variances at convolutional layer 1 (conv1) of the CNN where each response map is partitioned into $n \times n$ equally sized subregions. Responses were then recorded for each subregion and every filter by a max-pooling operation over the response maps. Two types of variances were calculated. First, we calculated the total variance over all 96 filter entries of the $n \times n$ subregions of conv1. This variance is henceforth called Variance $P_a(n)$ (Brachmann et al., 2017). Low values of this variance indicate that a large number of filters respond at many image positions (*richness* of filter responses). High values indicate few responses in a small number of subregions (*sparseness* of filter responses). The variances can be calculated for a different number of $n \times n$ subregions, which correspond to different levels of spatial resolution. In the present study, the number of subregions were 2×2, 4×4, 8×8, 16×16, and 30×30, resulting in variances henceforth called $P_a(2)$, $P_a(4)$, $P_a(8)$, $P_a(16)$, and $P_a(30)$. Second, we calculated the median over the variances of each of the 96 filters, again for different numbers of $n \times n$ subregions at conv1. This variance is henceforth termed Variance $P_f(n)$ and was calculated for the same number of subregions as Variance $P_a(n)$, resulting in variances henceforth called $P_f(2)$, $P_f(4)$, $P_f(8)$, $P_f(16)$, and $P_f(30)$. Low values of this variance indicate a high degree of self-similarity of filter responses across the response map, whereas high values indicate a greater variability of filter responses (Brachmann et al., 2017).

Traditional artworks of different cultural provenance show a particularly high richness of filter responses (i.e. low Variance $P_a[22]$ values) and a low to intermediate degree of variability (i.e., low Variance Pf[12] values; Brachmann et al., 2017). Because of the high degree of redundancy of the variances of each type, we used only one variance each in the final analysis ($P_a[2]$ and $P_f[30]$; Table 1).

1.4 Color Measures

Color is an important attribute of visual artworks and photographs (Li and Chen, 2009; Yanulevskaya et al., 2012; Rafegas and Vanrell, 2017). Artists seem to have an intuitive knowledge of chromatic image composition, which matches viewers' preferences, and implement this knowledge in their creations (Nascimento et al., 2017). Preferred color gamuts are perceived as natural (Nascimento et al., 2021) and colorful (Altmann et al., 2021). For abstract artworks, individual differences in color preferences have been reported (Mallon et al., 2014).

We measured nine chromatic features. For the L*a*b* color space, we included the mean values for each channel. For the HSV color space, we included both the mean values and the Shannon entropy for all three channels. In our final model, we retained three of the measures only to reduce collinearities: the mean value of the b channel (blue-yellow axis) of the L*a*b* color space (henceforth abbreviated Lab [b]); the mean value of the S-channel (saturation) of the HSV color space (HSV [S]); and the Shannon entropy of the H channel of the HSV color space (HSV [H] entropy; Table 1). HSV (H) entropy represents the uniformity of the color spectrum. Highest values are encountered if all hues are present at equal strength in the image. This measure thus represents a type of colorfulness.

1.5 Fourier Spectral Properties (Slope and Sigma)

The Fourier spatial frequency spectrum of natural and visually preferred images, such as artworks, shows statistical regularities (Graham and Field, 2007; Redies et al., 2007). Two characteristics of the spectrum, here called Fourier slope and Fourier sigma, were included in the initial set of 29 variables. The Fourier slope is the slope of log-log plots of spectral power versus spatial frequency and indicates the relative prominence of low versus high spatial frequencies (relation of coarse to fine structure) in an image. Grayscale images of natural scenes and traditional artworks fall off linearly with increasing spatial frequency and share a Fourier power slope of about -2 (Graham and Field, 2007; Redies et al., 2007). Fourier slope is correlated with Complexity (Table 1) because more shallow slopes (i.e., higher slope values) indicate more fine detail in an image. Fourier sigma is defined as the deviation of the Fourier spectral curve from a straight line in the log-log plots (Redies et al., 2007). It assumes small values for natural images and large sets of traditional artworks. When the deviation of the spectral curve from a straight line is large and Fourier sigma is high, images can be perceived as unpleasant (Fernandez and Wilkins, 2008).

1.6 Symmetry

The role of symmetry as an aesthetic primitive that is universally associated with visual preference (Bode et al., 2017) has recently been contested (Leder et al., 2019). In the present work, we calculated up-down mirror symmetry, left-right mirror symmetry as well as a combination of both symmetries, according to the algorithm described by (Brachmann and Redies, 2016). We use first-layer (conv1) filter responses of a CNN to measure higher-order symmetry. This measure relies not only on color, edges and texture depicted in an image, but also reflects the symmetry of simple

shapes and objects of an image, as does the human visual system. The three symmetry measures for the style-transferred images correlate moderately to highly with Self-similarity and Variance $P_f(30)$ (Spearman's coefficients $|\rho| = 0.61$ to 0.72), which represents the variability of filter responses (see above). Because of this redundancy and the contested role of symmetry as a universal mediator of beauty (Leder et al., 2019), the symmetry measures were not included in the final set of variables (Table 1).

Supplementary Figures

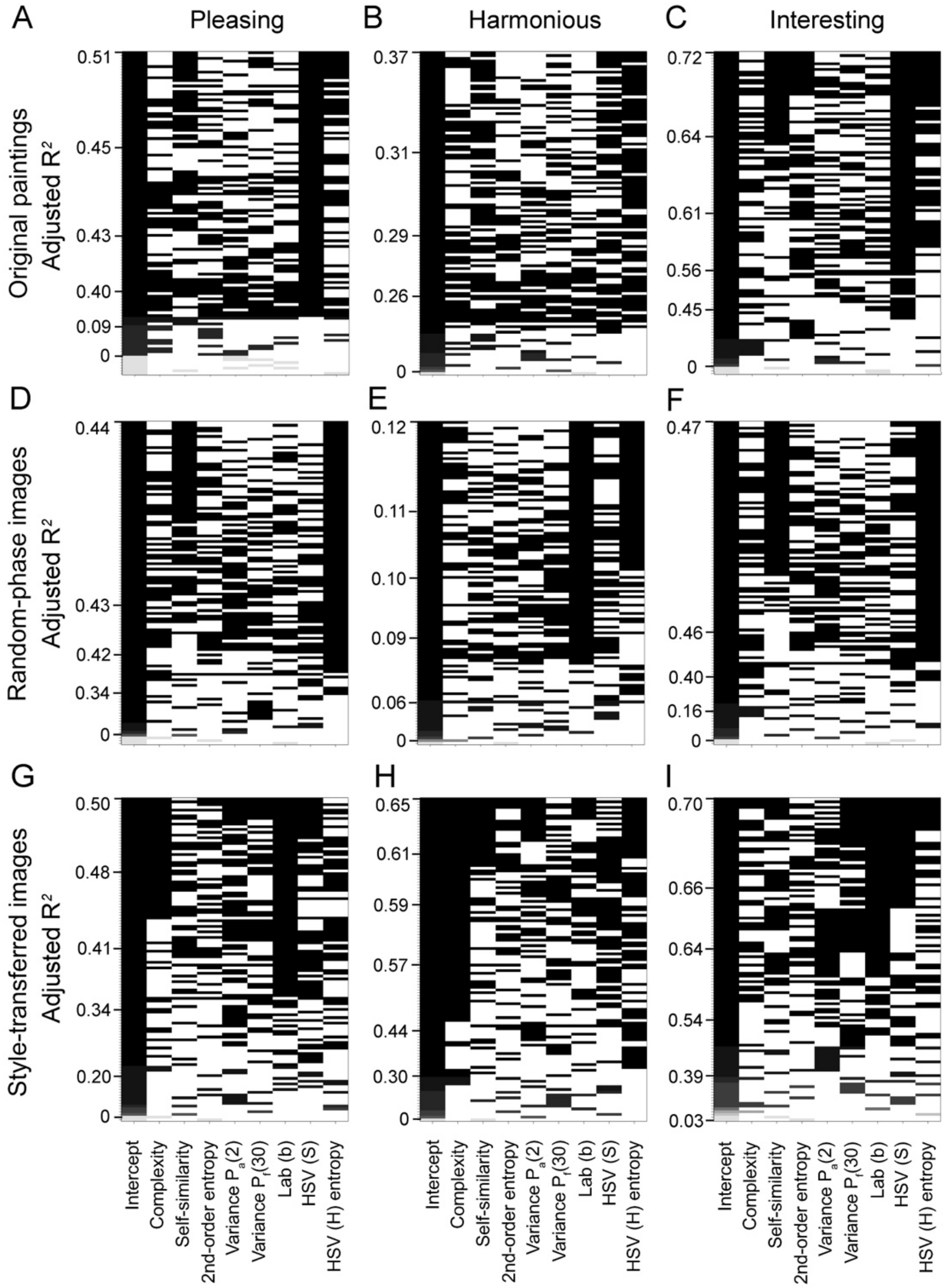

SUPPLEMENTARY FIGURE 1 | Results of regression subset selection for ratings of Pleasing (**A, D, G**), Harmonious (**B, E, H**), and Interesting (**C, F, I**). Data are shown for original artworks (**A – C**), random-phase images (**D – F**), and style-transferred images (**G – I**). Each horizontal line represents one regression model. The y-axis represents the R^2_{adj} value for each model. Model size was systematically varied from one SIP to all eight SIPs. The SIPs are indicated below the panels. For each model size, the 10 models with the highest R^2_{adj} values are displayed. The bars represent variables that are predictors in the model. The shadings of the bars represent the magnitude of the R^2_{adj} values for the models.

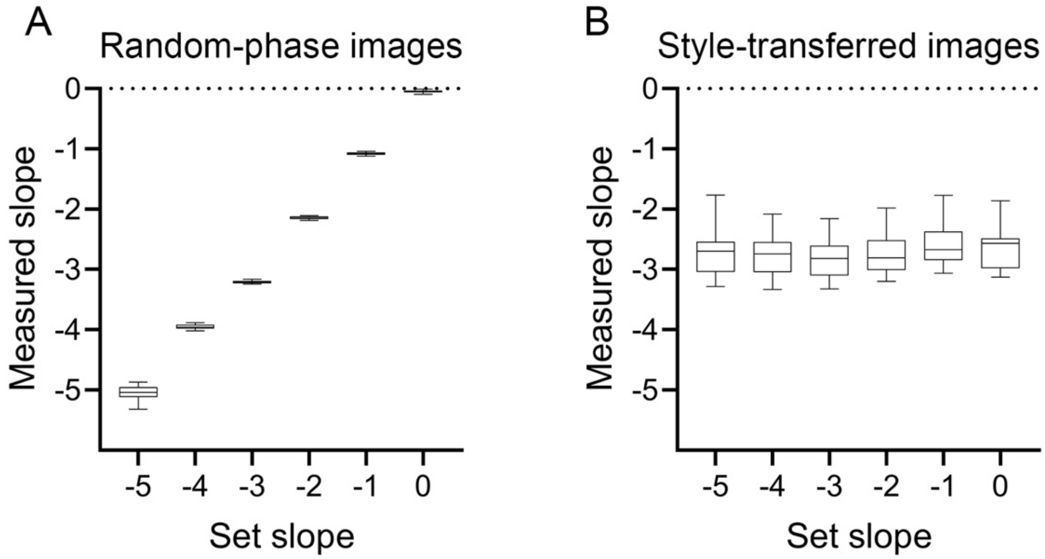

SUPPLEMENTARY FIGURE 2 | Comparison of the slope values, which were set during the generation of the random-phase images (x-axis), and the measured slope values (y-axis) for the 150 random-phase images (**A**; for examples, see Fig. **1D, F, H**) and the 150 derived style-transferred images (**B**; for examples, see **Fig. 1E, G, I**).

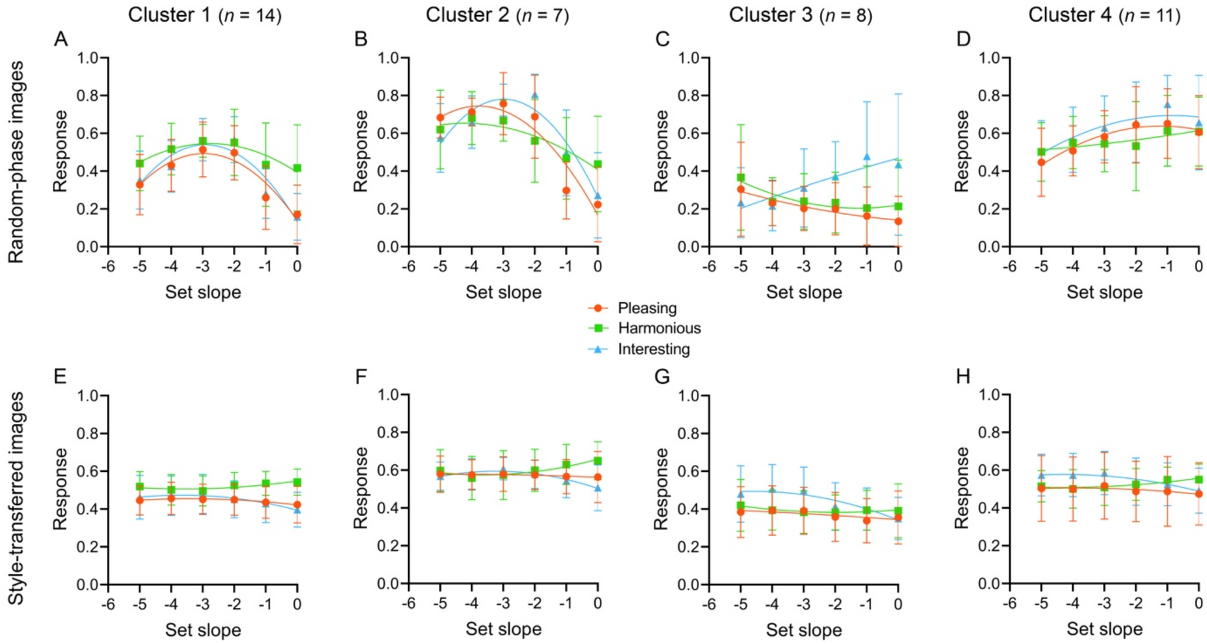

SUPPLEMENTARY FIGURE 3 | Mean rating responses by clusters of participants to different set Fourier spectral slopes (-5 to 0) of the random-phase images **(A – D)** and the style-transferred images **(E – H)**. The clustering was based on the mean rating responses to the random-phase images with different slope values. The curves represent least-squares fitting of second-order polynomial (quadratic) functions (*orange*, Pleasing; *green*, Harmonious; and *blue*, Interesting). *n*, number of participants in each cluster.

About half of the participants (Clusters 1 and 2) exhibit an inverted u-shaped response curve for all three rating dimensions, with the shallowest curve observed for Harmonious. For Clusters 3 and 4, curves appeared linear and a linear fitting was applied. For Cluster 3, we found a decrease for Pleasing (slope: -0.030, p<.05, F[1,46] = 5.56, 95% *CI*: -0.056 to -0.004), and an increase for Interesting (slope: 0.053, p<.05, F[1,64] = 7.21, *CI*: 0.013 to 0.093) with increasing set slope values. For Cluster 4, both Pleasing (slope: 0.037, p<.01, F[1,64] = 8.80, *CI*: 0.012 to 0.062) and Interesting (slope: 0.039, p<.01, F[1,64] = 8.06, *CI*: 0.011 to 0.066) increased. Slopes of the fitted lines were not significant for Harmonious for either cluster.

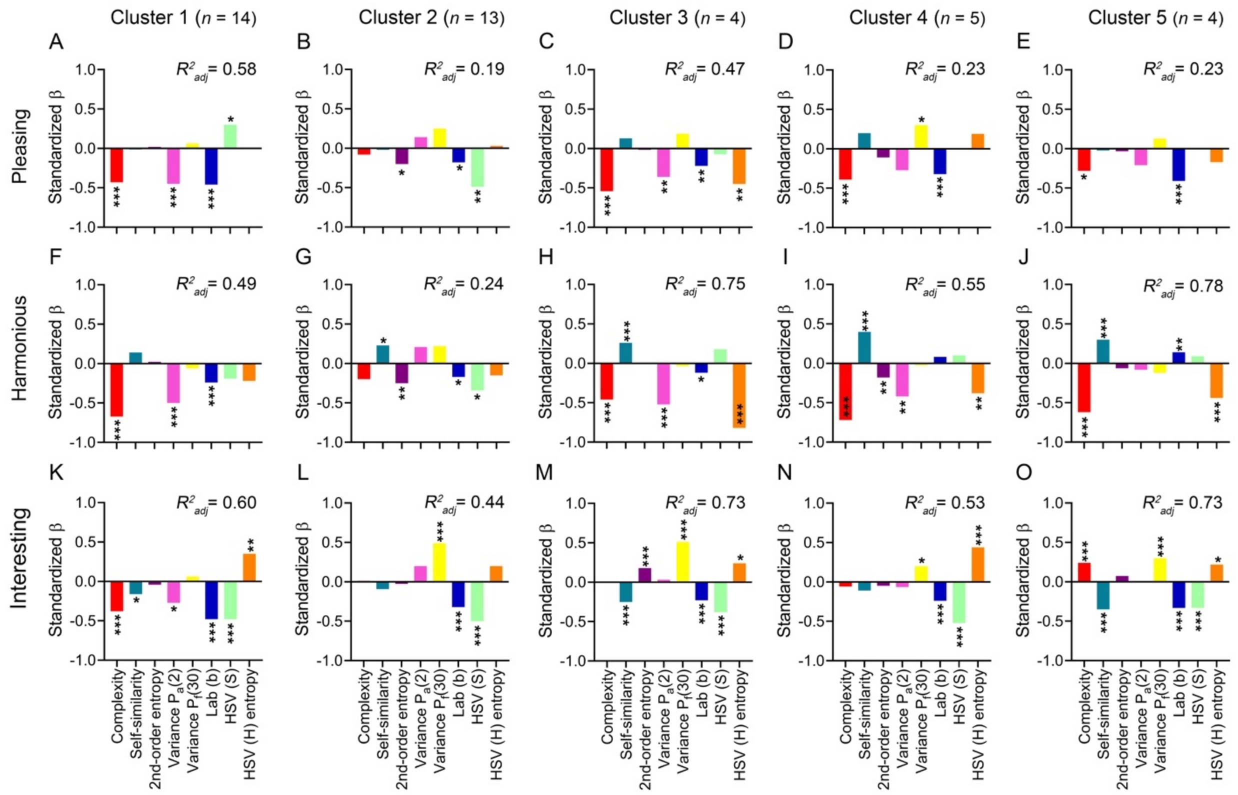

SUPPLEMENTARY FIGURE 4 | Standardized β* values for the influence of the statistical image properties (SIPs) on the rating responses in the style-transferred image set. Data are shown for the five clusters based on the correlations between the three rating dimensions (Pleasing, Harmonious, and Interesting). Data are visualized from **Supplementary Table 3**. Standardized β* values for the SIPs are shown separately for the five clusters (**A, F, K,** Cluster 1; **B, G, L,** Cluster 2; **C, H, M,** Cluster 3; **D, I, N,** Cluster 4; and **E, J, O,** Cluster 5). Results are shown for the three rating dimensions (**A – E**, Pleasing; **F – J**, Harmonious; and **K – O**, Interesting). The explained variance (R^2_{adj}) is indicated for each model (for all models, $p < .00001$). Asterisks indicate β* values of variables that had a significant effect on the ratings when the other variables were controlled for in the respective models; the respective significance levels are *, $p < .05$; **, $p < .01$; ***, $p < .001$.

SUPPLEMENTARY TABLES

Supplementary Table 1 | List of original paintings

1. **Albers**, Joseph (1888 – 1976). *Study for an Hommage to the Square*, 1965. 29.2 × 29.5 cm. 800 × 793 px. Constructivism, Hard-Edge Painting.
2. **Arp**, Jean (1886 – 1966). *Nez et Fauteuil*, 1925. 23 × 30.7 cm. 800 × 593 px. Dadaism.
3. **Baumeister**, Willi (1889 – 1955). *Bluxao V*, 1955. 130 × 100 cm. 630 × 800 px. Art Informel.
4. **Baziotes**, William (1912 – 1963). *Flame*, 1954. 106.7 × 89.2 cm. 680 × 800 px. Abstract Expressionism, Surrealism.
5. **Bissière**, Roger (1886 – 1964). *Composition 67 (Tache Verte)*, 1953. 31 × 39 cm. 800 × 626 px. Art Informel.
6. **Delaunay**, Robert (1885 – 1941). *Rythme, Joie de Vivre*, 1931. 203 × 180 cm. 703 × 800 px. Orphism.
7. **Frankenthaler**, Helen (1928 – 2011). *Tribal Sign*, 1987. 61 × 46.4 cm. 605 × 800 px. Abstract Expressionism.
8. **Hofmann**, Hans (1880 – 1966). *Bacchanale*, 1946. 162.6 × 122 cm. 588 × 800 px. Abstract Expressionism.
9. **Kandinsky**, Wassily (1866 – 1944). *Gelb-Rot-Blau*, 1925. 128 × 201.5 cm. 800 × 503 px. Abstract Expressionism, Constructivism.
10. **Klein**, Yves (1928 – 1962). *Monochrome Bleu*, 1961. 72 × 54 cm. 654 × 800 px. Monochrome Painting.
11. **Krasner**, Lee (1908 – 1984). *Polar Stampede*, 1960. 243.8 × 412.4 cm. 800 × 469 px. Abstract Expressionism.
12. **Manessier**, Alfred (1911 – 1953). *Les Fruits qui Tombent (Les Fruits Mûrs)*, 1949. 45.7 × 38.1 cm. 666 × 800 px. Tachism.
13. **Martinez**, Eddie (*1984). *Happy Painting*, 2014. 182.9 × 152.4 cm. 669 × 800 px, 181. Neo-Expressionism.
14. **Matta**, Roberto (1911 – 2002). *Esco Dalla Mia Rabbia*, 1964. 140.5 × 144 cm. 800 × 780 px. Abstract Expressionism.
15. **Moholy-Nagy**, László (1859 – 1946). *Z VII*, 1926. 95.3 × 76.2 cm. 657 × 800 px. Constructivism.
16. **Mondrian**, Piet (1872 – 1944). *Composition II in Red, Blue, and Yellow*, 1929. 59.5 × 59.5 cm. 796 × 800 px. Constructivism.
17. **Motherwell**, Robert (1915 – 1991). *Wall Painting No. III*, 1953. 137.2 × 184.2 cm. 800 × 619 px. Abstract Expressionism.
18. **Pollock**, Jackson (1912 – 1956). *Gothic*, 1944. 215.5 × 142.1 cm. 533 × 800 px. Abstract Expressionism.
19. **Riley**, Bridget (*1931). *Into Place*, 1987. 165.1 × 160 cm. 777 × 800 px. Hard-Edge Painting, Op Art.
20. **Rothko**, Mark (1903 – 1970). *Red on Maroon*, 1959. 266.7 × 238.8 cm. 722 × 800 px. Abstract Expressionism, Colour-Field Painting.
21. **Stella**, Frank (*1936). *Flin Flon XII*, 1970. 274.3 × 274.3 cm. 800 × 798 px. Abstract Expressionism, Hard-Edge Painting.
22. **Twombly**, Cy (1928 – 2011). *Nini's Painting*, 1971. 260.5 × 299.7 cm. 800 × 695 px. Abstract Expressionism.
23. **Vasarely**, Victor (1906 – 1997). *Folklore Planetaire*, ca. 1985. 53.5 × 53.5 cm. 780 × 800 px. Hard-Edge Painting, Op Art.
24. **WOLS** (1913 – 1951). *Untitled*, ca. 1940. 11.3 × 14.5 cm. 800 × 672 px. Art Informel.
25. **Wou-Ki**, Zao (1920 – 2013). 23.05.64, 1964. 200 × 162 cm. 645 × 800 px. Abstract Expressionism.

Supplementary Table 2 | R^2_{adj} values and β* values for all rating dimensions and image categories. The β* values indicate the influence of the statistical image properties (SIPs) on the rating responses. Asterisks for the R^2_{adj} values indicate significant models. The principal component analysis (PCA) model consisted of the first eight principal components (PCs) of all 29 variables. Asterisks with the β* values indicate statistical image properties (SIPs) that had a significant effect on the rating responses when the other variables were controlled for (in bold letters). Significance levels are *, p < .05; **, p < .01; ***, p < .001. Data are visualized in Figures 4 and 7.

		R^2_{adj} PCA Model with 8 PCs	R^2_{adj} 8 Variables	Complexity	Self-similarity	2nd-order entropy	$P_a(2)$	$P_f(30)$	Lab (b)	HSV (S)	HSV (H) entropy
Original paintings	Pleasing	0.36 *	0.40 *	-0.022	-0.47	0.080	-0.044	-0.28	-0.062	**-0.72 ***	0.40
	Harmonious	0.33	0.22	-0.15	-0.39	-0.025	-0.101	-0.29	0.064	-0.29	-0.30
	Interesting	0.61 **	0.67 ***	0.058	**-0.51 ***	0.33	-0.076	-0.18	-0.036	**-0.62 ****	0.45
Random-phase images	Pleasing	0.44 ***	0.42 ***	-0.18	0.72	-0.24	-0.023	-0.012	-0.057	-0.15	**1.05 ****
	Harmonious	0.11 **	0.096 **	-0.35	0.012	0.030	0.0096	-0.042	**-0.23 ****	-0.72	0.89
	Interesting	0.48 ***	0.46 ***	0.067	0.83	-0.19	0.16	-0.021	0.0092	-0.40	**1.06 ****
Style-transferred images	Pleasing	0.42 ***	0.50 ***	**-0.44 *****	0.043	-0.09	**-0.29 ***	**0.23 ***	**-0.43 *****	**-0.32 ***	-0.059
	Harmonious	0.59 ***	0.65 ***	**-0.68 *****	**0.31 *****	-0.095	**-0.35 ****	-0.0047	**-0.13 ***	-0.093	**-0.47 *****
	Interesting	0.69 ***	0.69 ***	-0.13	**-0.20 ****	0.0032	-0.050	**0.32 *****	**-0.41 *****	**-0.54 *****	**0.34 ****

Supplementary Table 3 | R^2_{adj} values and β* values for the five clusters that were obtained based on the inter-rating correlations. The β* values indicate the influence of the statistical image properties (SIPs) on the rating responses for the three rating dimensions. All model effect sizes (R^2_{adj}) were significant at a level of $p < .00001$. Asterisks with the β* values indicate variables that had a significant effect on the ratings when the other variables were controlled for (bold letters). Significance levels are *, $p < .05$; **, $p < .01$; ***, $p < .001$. Data are visualized in Supplementary Figure 4.

		R^2_{adj} 8 Variables	Complexity	Self-similarity	2nd-order entropy	$P_a(2)$	$P_f(30)$	Lab (b)	HSV (S)	HSV (H) entropy
Cluster 1 n = 14	Pleasing	0.58	**-0.43 *****	-0.013	0.019	**-0.45 *****	0.062	**-0.46 *****	**-0.30 ***	0.0070
	Harmonious	0.49	**-0.67 *****	0.14	0.022	**-0.50 *****	-0.061	**-0.24 *****	-0.19	-0.22
	Interesting	0.60	**-0.38 *****	**-0.16 ***	-0.040	**-0.27 ***	0.063	**-0.48 *****	**-0.48 *****	**0.35 ****
Cluster 2 n = 13	Pleasing	0.19	-0.080	-0.020	**-0.20 ***	0.14	0.25	**-0.18 ***	**-0.49 ****	0.030
	Harmonious	0.24	-0.20	**0.23 ***	**-0.25 ****	0.21	0.22	**-0.17 ***	**-0.34 ***	-0.15
	Interesting	0.44	0.013	-0.093	-0.026	0.20	**0.49 *****	**-0.32 *****	**-0.50 *****	0.20
Cluster 3 n = 4	Pleasing	0.47	**-0.54 *****	0.13	-0.016	**-0.36 ****	0.19	**-0.22 ****	-0.072	**-0.45 ****
	Harmonious	0.75	**-0.46 *****	**0.26 *****	0.0040	**-0.52 *****	-0.035	**-0.12 ***	0.18	**-0.82 *****
	Interesting	0.73	-0.0054	**-0.25 *****	**0.18 *****	0.031	**0.51 *****	**-0.23 *****	**-0.38 *****	**0.24 ***
Cluster 4 n = 5	Pleasing	0.23	**-0.39 *****	0.20	-0.11	-0.27	**0.30 ***	**-0.32 *****	-0.0041	0.19
	Harmonious	0.55	**-0.72 *****	**0.40 *****	**-0.18 ****	**-0.42 ****	-0.024	0.083	0.10	**-0.38 ****
	Interesting	0.53	-0.058	-0.11	-0.047	-0.065	**0.20 ***	**-0.24 *****	**-0.52 *****	**0.44 *****
Cluster 5 n = 4	Pleasing	0.23	**-0.28 ***	-0.023	-0.033	-0.21	0.13	**-0.41 *****	0.0070	-0.17
	Harmonious	0.78	**-0.62 *****	**0.30 *****	-0.063	-0.081	-0.12	**0.14 ****	0.089	**-0.44 *****
	Interesting	0.73	**0.24 *****	**-0.35 *****	0.075	-0.0073	**0.30 *****	**-0.33 *****	**-0.33 *****	**0.22 ***
